# RBM33 directs the nuclear export of transcripts containing GC-rich elements

**DOI:** 10.1101/gad.349456.122

**Published:** 2022-05-01

**Authors:** Anu Thomas, Frederick Rehfeld, He Zhang, Tsung-Cheng Chang, Mohammad Goodarzi, Frank Gillet, Joshua T. Mendell

**Affiliations:** 1 Department of Molecular Biology, University of Texas Southwestern Medical Center, Dallas, Texas 75390, USA;; 2 Quantitative Biomedical Research Center, University of Texas Southwestern Medical Center, Dallas, Texas 75390, USA;; 3 Department of Clinical Sciences, University of Texas Southwestern Medical Center, Dallas, Texas 75390, USA;; 4 Department of Immunology, University of Texas Southwestern Medical Center, Dallas, Texas 75390, USA;; 5 Harold C. Simmons Comprehensive Cancer Center, University of Texas Southwestern Medical Center, Dallas, Texas 75390, USA;; 6 Hamon Center for Regenerative Science and Medicine, University of Texas Southwestern Medical Center, Dallas, Texas 75390, USA;; 7 Howard Hughes Medical Institute, University of Texas Southwestern Medical Center, Dallas, Texas 75390, USA

**Keywords:** GC content, GC-rich elements, long noncoding RNAs, NORAD, nuclear export, RBM33, lncRNAs

## Abstract

Although splicing is a major driver of RNA nuclear export, many intronless RNAs are efficiently exported to the cytoplasm through poorly characterized mechanisms. For example, GC-rich sequences promote nuclear export in a splicing-independent manner, but how GC content is recognized and coupled to nuclear export is unknown. Here, we developed a genome-wide screening strategy to investigate the mechanism of export of *NORAD*, an intronless cytoplasmic long noncoding RNA (lncRNA). This screen revealed an RNA binding protein, RBM33, that directs the nuclear export of *NORAD* and numerous other transcripts. RBM33 directly binds substrate transcripts and recruits components of the TREX–NXF1/NXT1 RNA export pathway. Interestingly, high GC content emerged as the feature that specifies RBM33-dependent nuclear export. Accordingly, RBM33 directly binds GC-rich elements in target transcripts. These results provide a broadly applicable strategy for the genetic dissection of nuclear export mechanisms and reveal a long-sought nuclear export pathway for transcripts with GC-rich sequences.

## Abstract (precis)

In this study, Thomas et al. developed a genome-wide screening strategy to investigate the mechanism of export of *NORAD*, an intronless cytoplasmic long noncoding RNA (lncRNA), and found an RNA binding protein, RBM33, that directs the nuclear export of *NORAD* and numerous other transcripts. These results provide a broadly applicable strategy for the genetic dissection of nuclear export mechanisms and reveal a long-sought nuclear export pathway for transcripts with GC-rich sequences.

Nuclear export of RNA is a highly coordinated process that facilitates the selective transport of processed transcripts to the cytoplasm while retaining partially processed or misprocessed transcripts in the nucleus. This selectivity is achieved through the controlled deposition of specific proteins on an export-competent RNA following the completion of processing events such as capping, splicing, and polyadenylation (Heath et al. 2016). The canonical export pathway for messenger RNA in mammals relies on the transcription export (TREX) complex, composed of the hexameric THO subcomplex (THOC1–3 and THOC5–7), the DExD-box helicase UAP56 (also known as DDX39B), and the nuclear export factor ALYREF (Sträßer et al. 2002; Masuda et al. 2005; Chi et al. 2013). This complex is recruited to the mRNA via interactions of ALYREF with the cap binding complex (CBC) and the exon junction complex (EJC), deposited following capping and splicing, respectively (Le Hir et al. 2001; Cheng et al. 2006; Singh et al. 2012; Chi et al. 2013; Viphakone et al. 2019). ALYREF, in conjunction with 3′ end processing factors, then recruits the NXF1–NXT1 heterodimer, the nuclear export receptor that binds the RNA and carries it through the nuclear pore complex into the cytoplasm (Hautbergue et al. 2008; Katahira et al. 2009; Viphakone et al. 2012).

Although transcripts that use the TREX pathway for nuclear export generally require splicing for efficient export (Katahira 2012), unspliced transcripts constitute ∼5% of the human transcriptome (Shabalina et al. 2010). Our understanding of the mechanisms that promote nuclear export of most intronless RNAs remains limited. Viral RNAs have served as important models for investigating the export of unspliced transcripts, and their study has revealed elegant mechanisms by which this class of RNAs hijacks nuclear export pathways (Sandri-Goldin 2004). For example, the human immunodeficiency virus type-1 (HIV-1) REV protein binds to a *cis*-element called the REV response element (RRE) in incompletely spliced viral RNAs, routing them through the CRM1-dependent protein export pathway (Fornerod et al. 1997; Neville et al. 1997). A highly structured *cis*-element in unspliced mRNAs encoded by the Mason–Pfizer monkey virus (MPMV), termed the constitutive transport element (CTE), directly recruits NXF1, obviating the need for additional *trans*-acting factors to promote nuclear export (Ernst et al. 1997; Braun et al. 1999). In keeping with these export strategies used by viral RNAs, multiple studies have reported the existence of *cis*-elements in naturally intronless mammalian mRNAs, termed cytoplasmic accumulation regions (CARs), that mediate RNA export via the NXF1 pathway (Lei et al. 2011, 2013). In addition, high GC content, especially at the 5′ end, has been shown to promote the nuclear export of unspliced transcripts, although how GC-rich elements recruit the nuclear export machinery is unknown (Palazzo et al. 2007; Cenik et al. 2011; Palazzo and Akef 2012; Mordstein et al. 2020; Zuckerman et al. 2020).

Long noncoding RNAs (lncRNAs) are a class of non-protein-coding transcripts >200 nt in length with diverse functions (Kopp and Mendell 2018). Owing to the early discovery of several nuclear lncRNAs, this class of transcripts was initially believed to be enriched in the nucleus. Subsequent identification and characterization of many lncRNAs, however, has led to the appreciation that these transcripts are broadly distributed throughout the cell (Ulitsky and Bartel 2013; Carlevaro-Fita and Johnson 2019). While sequence elements that lead to nuclear retention of lncRNAs have been identified (Lubelsky and Ulitsky 2018; Shukla et al. 2018), mechanisms governing lncRNA trafficking to other subcellular locations remain poorly characterized.

*Noncoding RNA activated by DNA damage* (*NORAD*) is a prominent example of a lncRNA whose precise subcellular localization is essential for its function. This 5.3-kb unspliced lncRNA is efficiently exported to the cytoplasm, where it binds and sequesters Pumilio (PUM) RNA binding proteins in condensates termed *NORAD*-Pumilio (NP) bodies (Lee et al. 2016; Tichon et al. 2016; Elguindy et al. 2019; Kopp et al. 2019; Elguindy and Mendell 2021). PUM proteins are cytoplasmic RNA binding proteins that post-transcriptionally repress target mRNAs (Miller and Olivas 2011; Quenault et al. 2011). Because PUM targets are enriched for regulators of mitosis, DNA replication, and DNA repair, loss of *NORAD* and/or PUM hyperactivity leads to a dramatic genomic instability phenotype in mammalian cells and premature aging in mouse models (Lee et al. 2016; Tichon et al. 2016; Kopp et al. 2019; Elguindy and Mendell 2021). Efficient nuclear export of *NORAD*, which is critical for its function, is dependent on NXF1 and TPR, a nuclear pore component that functions downstream from NXF1 (Lee et al. 2020; Zuckerman et al. 2020). GC-rich sequences at the 5′ end of *NORAD* were shown to be sufficient to promote NXF1-dependent export of an unspliced reporter transcript (Zuckerman et al. 2020). Nevertheless, it remains unclear whether these sequence elements are essential for *NORAD* export and how such sequences efficiently recruit NXF1 to initiate transport to the cytoplasm.

To identify factors required for the nuclear export of *NORAD* and potentially other intronless transcripts, we carried out a genome-wide CRISPR–Cas9 loss-of-function screen using a reporter cell line in which a green fluorescent protein (GFP) open reading frame, inserted into the endogenous *NORAD* locus, is translated only when *NORAD* is exported to the cytoplasm. In addition to known RNA export factors, this screen revealed a poorly characterized RNA binding protein, RBM33, that promotes the nuclear export of *NORAD*, thereby preventing its degradation by the nuclear exosome. RBM33 directs *NORAD* nuclear export by directly binding to this lncRNA and recruiting UAP56 and NFX1. RNA-seq of RBM33-deficient cell lines identified hundreds of additional transcripts whose efficient nuclear export requires RBM33. Surprisingly, RBM33 substrates were not enriched for intronless RNAs. Rather, high GC content emerged as the most prevalent feature of transcripts that depend on RBM33 for nuclear export. Accordingly, transcriptome-wide mapping of RBM33 binding sites using enhanced cross-linking immunoprecipitation (eCLIP) demonstrated binding to GC-rich elements. These results introduce a flexible screening approach for the genetic interrogation of nuclear RNA export mechanisms and uncover an RBM33-dependent RNA export route that promotes the cytoplasmic localization and enhanced expression of GC-rich RNAs.

## Results

### A CRISPR–Cas9 screen identifies factors required for *NORAD* nuclear export

**Figure 1.**
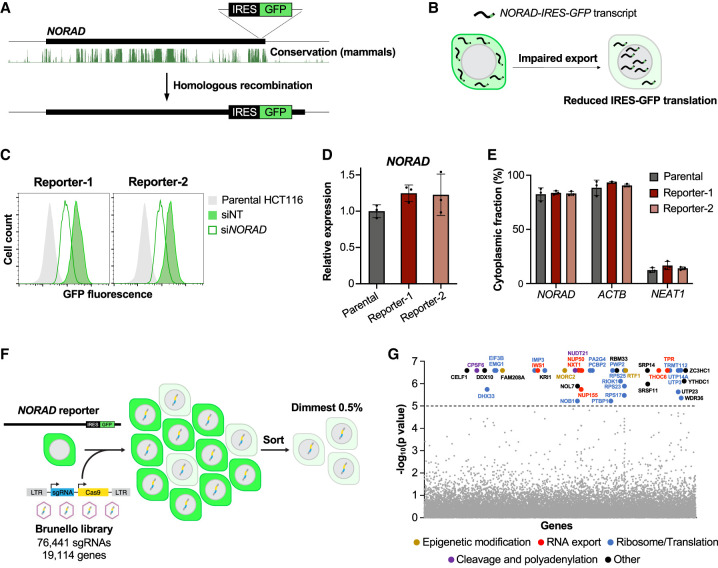
A genome-wide CRISPR–Cas9 screen for *NORAD* localization factors. (*A*) *NORAD* reporter cell lines were generated by the insertion of an IRES-GFP cassette into the endogenous *NORAD* locus. UCSC genome browser track depicting mammalian conservation (PhastCons, hg38) is shown. (*B*) Schematic representation of reporter cells upon the loss of a putative nuclear export factor for *NORAD*. (*C*) Flow cytometry analysis of GFP expression in two independent *NORAD* reporter clones following transfection of the indicated siRNAs. (NT) Nontarget control. (*D*,*E*) qRT-PCR analysis of *NORAD* expression relative to 18S rRNA (*D*) and cytoplasmic localization (*E*) in parental and *NORAD* reporter HCT116 cells. *ACTB* and *NEAT1* represent cytoplasmic and nuclear controls, respectively. (*F*) Overview of the CRISPR–Cas9 screen. (LTR) Long terminal repeat, (sgRNA) single guide RNA. (*G*) MAGeCK analysis of screening results. Genes, color coded by functional category, are plotted by MAGeCK *P*-value. For qRT-PCR experiments, data are represented as mean ± SD with individual data points shown (*n* = 3 biological replicates).

We developed a genome-wide CRISPR–Cas9 screening strategy to enable the unbiased identification of factors that are required for the nuclear export of *NORAD*. Using homologous recombination, we first inserted an internal ribosome entry site coupled to a GFP open reading frame (IRES-GFP) into the endogenous *NORAD* locus, near the 3′ end of the transcript, in the stably diploid cell line HCT116 (Fig. 1A). We hypothesized that the *NORAD-IRES-GFP* transcript would provide a readout of *NORAD* localization, since GFP can only be translated after transport of the transcript to the cytoplasm. Thus, cells with loss of function of factors that are essential for *NORAD* trafficking would exhibit reduced IRES-GFP translation and a corresponding decrease in fluorescence (Fig. 1B).

Two independent *NORAD-IRES-GFP* reporter cell lines were generated using distinct single guide RNAs (sgRNAs). Northern blotting confirmed that the full-length *NORAD* transcript exhibited the expected increase in size in *NORAD-IRES-GFP* reporter clones (Supplemental Fig. S1A), while flow cytometry demonstrated that reporter cells displayed ∼10-fold higher GFP fluorescence compared with parental HCT116 cells (Supplemental Fig. S1B). Moreover, short interfering RNA (siRNA)-mediated knockdown of *NORAD* confirmed that fluorescence intensity was coupled to *NORAD* abundance in reporter cells (Fig. 1C; Supplemental Fig. S1C). Importantly, insertion of the IRES-GFP cassette did not affect *NORAD* expression or cytoplasmic localization, suggesting that this reporter system could be used as a reliable readout for *NORAD* trafficking (Fig. 1D,E).

*NORAD* reporter cells were then infected with the Brunello lentiviral CRISPR library targeting ∼19,000 human genes (Doench et al. 2016). Twelve days after transduction, the dimmest 0.5% of cells, expected to be enriched for sgRNAs targeting *NORAD* localization factors, were collected by fluorescence-activated cell sorting (FACS) (Fig. 1F). sgRNA representation in sorted and unsorted populations was determined by high-throughput sequencing, and genes targeted by sgRNAs enriched in sorted cells were identified and ranked by model-based analysis of genome-wide CRISPR–Cas9 knockout (MAGeCK) (Fig. 1G; Supplemental Table S1; Li et al. 2014). Application of a stringent cutoff (*P* < 10^−5^) yielded a large number of potential regulators of the *NORAD-IRES-GFP* reporter (Fig. 1G; Supplemental Fig. S2A).

Many of the hits encoded proteins with functions related to ribosome biogenesis or translation, including multiple components of the small subunit processome, which mediates biogenesis of 18S rRNA (*UTP3*, *UTP14A*, and *UTP23*) (Dragon et al. 2002) and several small ribosomal subunit proteins (*RPS17*, *RPS23*, and *RPS25*). It is probable that the loss of these genes resulted in decreased fluorescence of the *NORAD* reporter through their effect on IRES-driven translation, rather than by influencing *NORAD* localization or abundance. IRES-dependent translation is generally less efficient than canonical cap-dependent translation and is therefore likely to be highly sensitive to depletion of ribosomal subunits. Genes that encode epigenetic regulators and components of the cleavage and polyadenylation machinery were also identified as hits, presumably due to their impact on *NORAD* transcription and 3′ end processing. Reassuringly, several RNA export factors, including *TPR*, which is known to be required for cytoplasmic trafficking of *NORAD* (Lee et al. 2020), were recovered as significant hits, establishing that this screening platform can identify factors that facilitate *NORAD* nuclear export.

Among the remaining highly ranked hits were several that encoded putative or validated RNA binding proteins (*RBM33*, *CELF1*, *DDX10, YTHDC1*, *NOL7*, *SRP14*, and *SRSF11*), raising the possibility that one or more of these factors may play a direct role in *NORAD* expression or trafficking. We assessed the effect of knocking out each of these genes, as well as *ZC3HC1*, another highly ranked hit that is required for TPR localization to the nuclear pore complex (Gunkel et al. 2021), on GFP expression, *NORAD* expression, and *NORAD* localization by generating knockout pools with lentivirally delivered CRISPR components (Supplemental Fig. S2B). As a control, we targeted the general poly(A) RNA export factor *IWS1* (Yoh et al. 2007), a highly ranked hit from the screen whose loss resulted in decreased GFP fluorescence and strong nuclear accumulation of both *NORAD* and *ACTB* (Supplemental Fig. S2B). We hypothesized that a factor required for splicing-independent export of *NORAD* would result in selective trapping of this lncRNA in the nucleus while minimally impacting export of *ACTB*. Among the validated genes, loss of *RBM33*, the top-ranked hit in the screen, resulted in the strongest nuclear enrichment of *NORAD* and associated reduction in GFP fluorescence without affecting *ACTB* trafficking. Moreover, *RBM33* knockout led to a strong decrease in *NORAD* abundance, possibly resulting from an increased susceptibility to degradation by the nuclear exosome, a phenomenon known to occur upon impaired nuclear export of intronless transcripts and other classes of RNAs (Fan et al. 2017). Other validated hits, including *ZC3HC1*, *SRP14*, and *SRSF11*, also appeared to regulate *NORAD* abundance and/or trafficking, albeit to a lesser extent. Given that RBM33 is a poorly characterized protein with a predicted RNA recognition motif whose loss of function had the strongest effect on the abundance and nuclear enrichment of *NORAD*, we decided to further study its role as a potential RNA export factor for *NORAD*.

### RBM33 is required for *NORAD* expression and nuclear export

**Figure 2.**
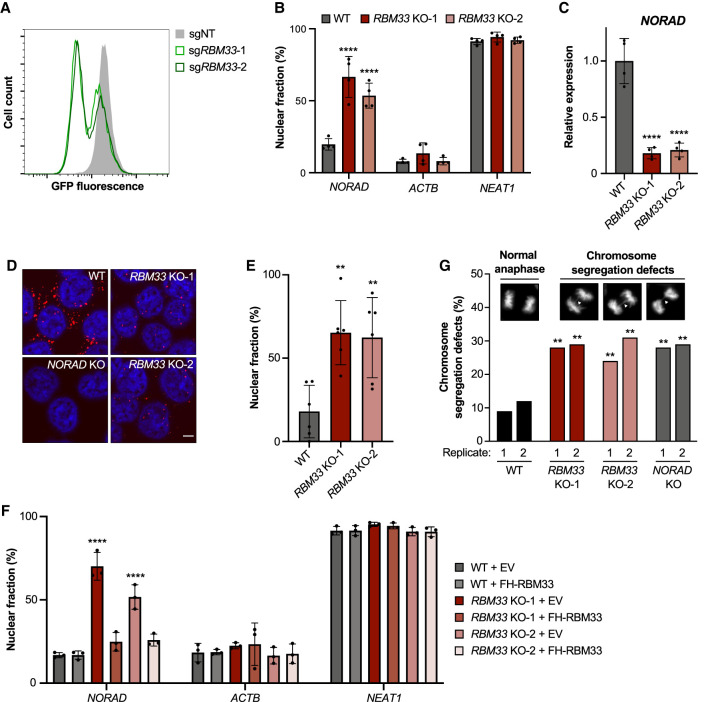
Loss of RBM33 results in nuclear enrichment and decreased abundance of *NORAD.* (*A*) Flow cytometry analysis of GFP expression in *NORAD* reporter cells following lentiviral delivery of Cas9 and nontarget (NT) control sgRNA or two independent sgRNAs targeting *RBM33*. (*B,C*) qRT-PCR analysis of the fraction of *NORAD*, *ACTB*, and *NEAT1* in the nucleus (*B*) or *NORAD* expression relative to 18S rRNA (*C*) in WT and *RBM33* KO HCT116 clones. (*D*) *NORAD* RNA FISH in HCT116 cells of the indicated genotypes. (Red) *NORAD* FISH, (blue) DAPI. Scale bar, 5 µm. (*E*) Quantification of the percentage of nuclear-localized *NORAD* FISH signal. Each data point represents a field containing at least 20 cells. (*F*) qRT-PCR analysis of the fraction of *NORAD*, *ACTB*, and *NEAT1* in the nucleus of WT and *RBM33* KO clones following lentiviral expression of FLAG-HA-RBM33 (FH-RBM33) or empty vector (EV) control. (*G*) Analysis of mitotic segregation defects in cells of the indicated genotypes. The *inset* shows representative images of anaphase cells with normal and defective chromosome segregation in DAPI-stained *RBM33* KO-2 cells. qRT-PCR and FISH data are represented as mean ± SD with individual data points shown. For qRT-PCR experiments, *n* = 3–4 biological replicates, and *P*-values were calculated by one-way ANOVA (*C*,*E*) or two-way ANOVA (*B*,*F*) comparing *RBM33* KO clones with WT (*B*,*C*,*E*) or each condition with WT + EV (*F*). For *G*, *P*-values were calculated by χ^2^ test comparing all replicate 1 samples with WT replicate 1 and all replicate 2 samples with WT replicate 2. (**) *P* ≤ 0.01, (****) *P* ≤ 0.0001.

Depletion of RBM33 using two distinct sgRNAs similarly reduced expression of the *NORAD-IRES-GFP* reporter, further validating the screening results (Fig. 2A). To verify that regulation of *NORAD* expression and trafficking by RBM33 was independent of the IRES-GFP cassette, we depleted RBM33 in unmodified HCT116 cells using siRNA (Supplemental Fig. S3A). As observed following CRISPR-mediated knockout, RBM33 knockdown resulted in strong nuclear enrichment of *NORAD* (Supplemental Fig. S3B) with a concomitant decrease in overall *NORAD* levels (Supplemental Fig. S3C).

To further study the role of RBM33 in *NORAD* expression and trafficking, two clonal HCT116 *RBM33* knockout cell lines were generated using distinct sgRNAs. Although both clones harbored homozygous out-of-frame indels, *RBM33* KO-1 exhibited frame-restoring alternative splicing of mutant transcripts, resulting in the production of an internally deleted RBM33 protein (Supplemental Fig. S3D,E). *RBM33* KO-2 displayed a near-complete loss of RBM33 protein. Several lines of evidence demonstrated that both of these mutations resulted in RBM33 loss of function. Both *RBM33* knockout clones exhibited strong nuclear enrichment of *NORAD* (Fig. 2B) accompanied by a significant decrease in total *NORAD* levels (Fig. 2C), closely mirroring the phenotype observed in CRISPR knockout pools and in cells depleted of RBM33 by siRNA. RNA fluorescence in situ hybridization (FISH) further documented nuclear localization and reduced overall expression of *NORAD* in *RBM33* knockout cells (Fig. 2D,E). Additionally, lentiviral expression of FLAG-HA-tagged RBM33 (FH-RBM33) at near-endogenous levels in *RBM33* knockout cells (Supplemental Fig. S3F) rescued *NORAD* localization (Fig. 2F) and increased *NORAD* abundance (Supplemental Fig. S3G), demonstrating that the observed phenotypes were due to RBM33 loss of function. Notably, *RBM33* knockout cells exhibited a high frequency of chromosome segregation defects (Fig. 2G), consistent with our prior demonstration that *NORAD* depletion results in genomic instability (Lee et al. 2016; Elguindy et al. 2019; Kopp et al. 2019; Elguindy and Mendell 2021).

**Figure 3.**
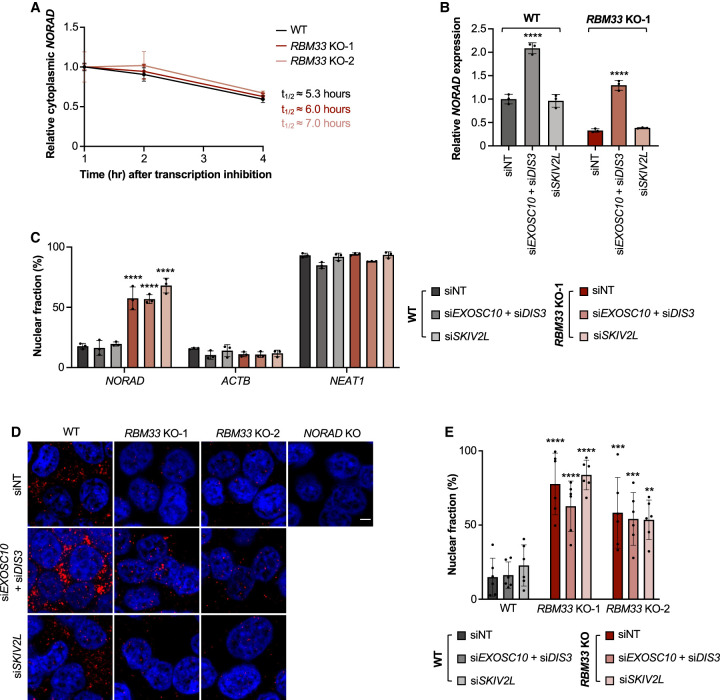
Loss of RBM33 impairs *NORAD* nuclear export, resulting in degradation by the nuclear exosome. (*A*) qRT-PCR analysis of *NORAD* cytoplasmic decay rates in HCT116 cells of the indicated genotypes. *NORAD* levels were normalized to *ACTB* abundance at each time point. (*B*) qRT-PCR analysis of *NORAD* expression relative to 18S rRNA in WT and *RBM33* KO-1 cells transfected with the indicated siRNA Smartpools. *P*-values were calculated by one-way ANOVA comparing each condition with nontarget (NT) siRNA in the same genotype. (*C*) The fraction of *NORAD*, *ACTB*, and *NEAT1* in the nucleus in WT and *RBM33* KO-1 cells transfected with the indicated siRNA Smartpools. *P*-values were calculated by two-way ANOVA comparing each condition with siNT in WT cells. (*D*) *NORAD* RNA FISH in HCT116 cells of the indicated genotypes transfected with the indicated siRNA Smartpools. (Red) *NORAD* FISH, (blue) DAPI. Scale bar, 5 µm. (*E*) Quantification of the percentage of nuclear-localized *NORAD* FISH signal. Each data point represents a field containing at least 20 cells. Data are represented as mean ± SD with individual data points shown. *P*-values were calculated by one-way ANOVA comparing each condition with WT transfected with siNT. For qRT-PCR experiments, *n* = 3 biological replicates. (**) *P* ≤ 0.01, (***) *P* ≤ 0.001, (****) *P* ≤ 0.0001.

The observed effects of RBM33 deficiency on *NORAD* expression and localization could be the result of impaired nuclear export and concomitant degradation by the nuclear exosome. Alternatively, cytoplasmic decay of *NORAD* in RBM33-deficient cells could give rise to a similar phenotype by specifically depleting the cytoplasmic *NORAD* pool. To distinguish between these possibilities, we first measured the stability of cytoplasmic *NORAD* transcripts in wild-type and *RBM33* knockout cells following transcription inhibition with actinomycin D. Decay of cytoplasmic *NORAD* was not accelerated in RBM33-deficient cells (Fig. 3A), a finding most consistent with nuclear trapping and subsequent degradation of *NORAD* by the nuclear exosome upon loss of RBM33. To further examine this possibility, nuclear and cytoplasmic RNA exosome components were depleted, and the effects on *NORAD* expression and localization were assessed. Loss of function of the nuclear exosome, achieved by the combined knockdown of EXOSC10 and DIS3 (Morton et al. 2018), resulted in an increase in *NORAD* abundance in both wild-type and *RBM33* knockout cells (Fig. 3B; Supplemental Fig. S4A,B,D,E). In contrast, inhibition of the cytoplasmic exosome by depletion of SKIV2L had no effect on *NORAD* levels. Importantly, cellular fractionation and RNA FISH demonstrated that the increased pool of *NORAD* in EXOSC10/DIS3-depleted cells was efficiently exported to the cytoplasm in wild-type cells, but remained trapped in the nucleus in *RBM33* knockout cells (Fig. 3C–E; Supplemental Fig. S4C). Consistent with its minimal effect on *NORAD* abundance, loss of SKIV2L did not impact *NORAD* localization. These results provide strong evidence in support of nuclear trapping and degradation of *NORAD* by the nuclear exosome in RBM33-deficient cells. Our finding that *NORAD* abundance is regulated by EXOSC10/DIS3 in wild-type cells is in keeping with the previously reported observation that the steady-state abundance of many transcripts, particularly those lacking introns, is regulated by competition between nuclear export pathways and degradation by the nuclear exosome (Fan et al. 2017).

### RBM33 directly links *NORAD* to the TREX–NXF1 nuclear export machinery

**Figure 4.**
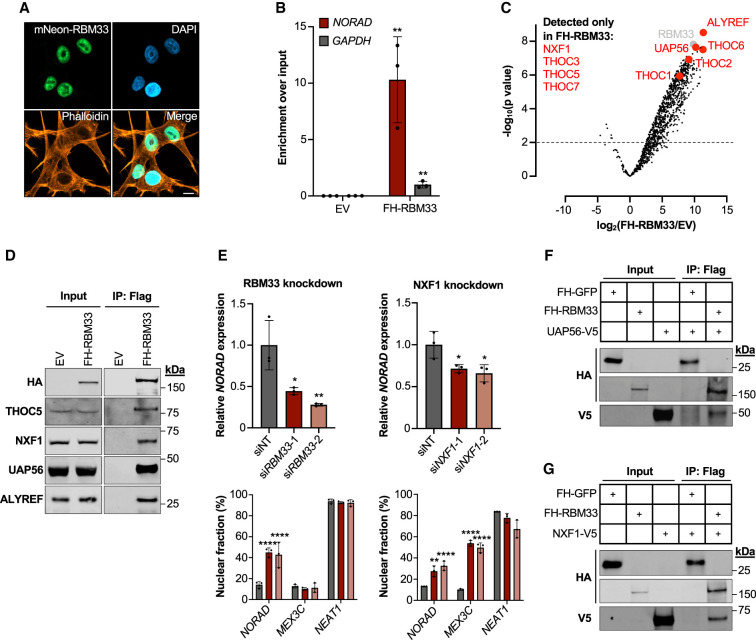
RBM33 promotes nuclear export via the TREX/NXF1 pathway. (*A*) Localization of endogenously tagged RBM33 in HCT116 cells. (Green) mNeonGreen, (orange) phalloidin, (blue) DAPI. Scale bar, 10 µm. (*B*) qRT-PCR analysis of *NORAD* and *GAPDH* in UV-RIP samples after pull-down of FH-RBM33 or empty vector (EV) control. For each sample, enrichment was first normalized to input and then normalized to *GAPDH* in FH-RBM33 pull-downs. (*C*) Volcano plot showing fold change and significance of proteins copurifying with FH-RBM33 and detected by mass spectrometry compared with EV control. Pull-downs were performed in the presence of RNase. TREX components and NXF1 are highlighted in red. (*D*) Western blot of coimmunoprecipitation of endogenous THOC5, NXF1, UAP56, or ALYREF with FH-RBM33. (*E*) qRT-PCR analysis of *NORAD* expression (*top*) and *NORAD*, *MEX3C*, and *NEAT1* localization (*bottom*) in HCT116 cells following siRNA-mediated knockdown of the indicated proteins. *MEX3C*, whose export is NXF1-dependent, served as a positive control. (*F*,*G*) Coimmunoprecipitation of in vitro translated FH-RBM33 and V5-tagged UAP56 (*F*) or NXF1 (*G*) detected by Western blot. FH-GFP served as a negative control. Data are represented as mean ± SD with individual data points shown. For qRT-PCR experiments, *n* = 3 biological replicates, and *P*-values were calculated by Student's *t*-test (*B*), one-way ANOVA (*E*, *top*), or two-way ANOVA (*E*, *bottom*). (*) *P* ≤ 0.05, (**) *P* ≤ 0.01, (****) *P* ≤ 0.0001.

Based on these data, we hypothesized that RBM33 may direct the nuclear export of *NORAD* by directly binding to this transcript and promoting its trafficking to the cytoplasm, thereby preventing its decay by the nuclear exosome. Like other nuclear export factors (Zhou et al. 2000; Hautbergue et al. 2009), endogenously tagged RBM33 (Fig. 4A) or heterologously expressed FH-RBM33 (Supplemental Fig. S5A) localized predominantly to the nucleus. Moreover, heterokaryon assays demonstrated that RBM33 shuttled between the nucleus and cytoplasm (Supplemental Fig. S5B). Additionally, UV cross-linking and RNA immunoprecipitation (UV-RIP) with FH-RBM33 resulted in strong and specific enrichment of *NORAD*, consistent with direct binding of RBM33 to this lncRNA (Fig. 4B; Supplemental Fig. S6A).

Since genes that encode several components of the canonical TREX-dependent RNA export pathway, including *NXT1* and *THOC6* (Carmody and Wente 2009), were recovered as significant hits in the screen, we speculated that RBM33 enables RNA export by linking its substrates to export factors in this pathway. To investigate this possibility, immunoprecipitation of FH-RBM33 from RNase-treated lysates followed by mass spectrometry was performed to identify RMB33-interacting proteins. Indeed, all core TREX components, including THOC1–3, THOC5–7, UAP56, ALYREF, and NXF1, were highly enriched in FH-RBM33 pull-downs (Fig. 4C; Supplemental Table S2). Coimmunoprecipitation of FH-RBM33 with THOC5, NXF1, UAP56, and ALYREF was confirmed by Western blotting (Fig. 4D).

These interaction data implicate the TREX–NXF1 pathway in RBM33-dependent nuclear export, which is consistent with the previously established role of NXF1 in *NORAD* trafficking (Zuckerman et al. 2020). Knockdown of NXF1 confirmed its requirement for *NORAD* expression and export (Fig. 4E; Supplemental Fig. S6B). Surprisingly, however, loss of ALYREF did not impair *NORAD* expression or result in its nuclear accumulation (Supplemental Fig. S6C), suggesting that this core TREX component is not required for *NORAD* export.

To identify specific components of the TREX complex that interact with RBM33, binding assays using in vitro translated FH-RBM33 and V5-tagged THO complex subunits (THOC1, THOC3, THOC5, and THOC7), UAP56, or NXF1 were performed. Although no detectable interactions were observed between RBM33 and any of the tested THO subunits (Supplemental Fig. S6D), these assays revealed interactions of RBM33 with UAP56 and NXF1 (Fig. 4F,G). Altogether, these genetic and biochemical experiments demonstrated that RBM33 promotes the nuclear export of *NORAD* by binding directly to this transcript and recruiting UAP56 and NXF1, thereby facilitating transport to the cytoplasm independently of splicing and ALYREF.

### RBM33 loss of function impairs nuclear export of GC-rich transcripts

Having established an essential role for RBM33 in the nuclear export of *NORAD*, we next investigated whether it is required for cytoplasmic transport of other RNAs. Unlike knockdown of ALYREF, which results in the nuclear accumulation of bulk poly(A)^+^ RNA, depletion of RBM33 did not impact overall poly(A)^+^ RNA export (Supplemental Fig. S7), suggesting that it may promote the cytoplasmic transport of a select clientele.

**Figure 5.**
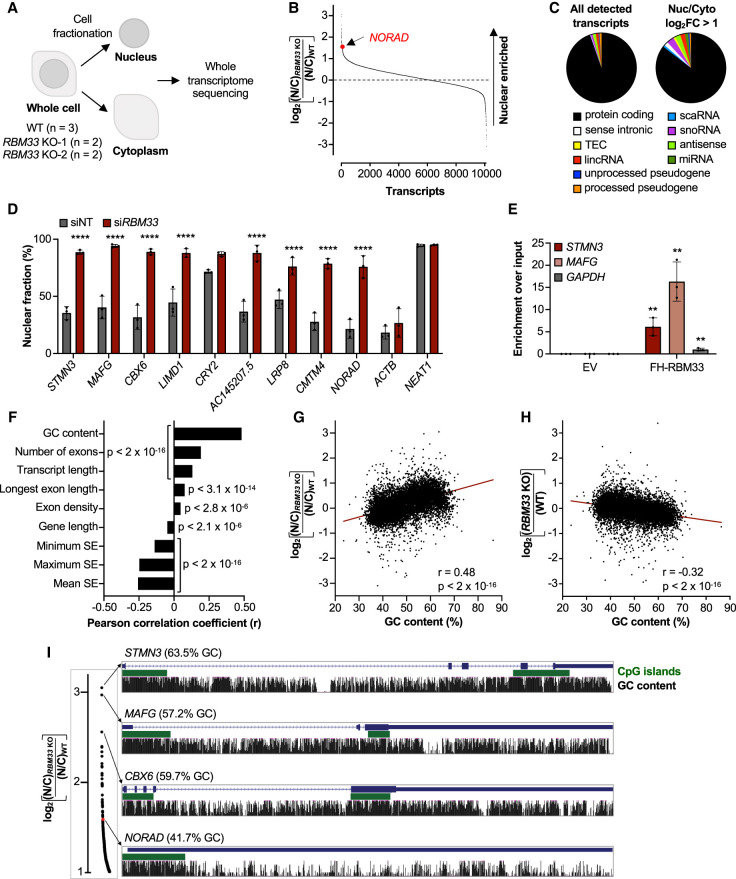
Loss of RBM33 impairs nuclear export of GC-rich transcripts. (*A*) Schematic representation of the RNA fractionation sequencing experiment. (*B*) Log_2_ fold changes of the nuclear to cytoplasmic ratios of transcripts in *RBM33* KO cells relative to WT HCT116 cells. (*C*) GENCODE biotypes of all detected transcripts and those exhibiting a log_2_ fold change (FC) >1 in their nuclear to cytoplasmic ratios in *RBM33* KO cells. (*D*) qRT-PCR analysis of the nuclear fraction of selected transcripts in *RBM33-*depleted cells. The *NORAD*, *ACTB,* and *NEAT1* data are also shown in Supplemental Figure S3B. (*E*) qRT-PCR analysis of *STMN3*, *MAFG*, and *GAPDH* in UV-RIP samples after pull-down of FH-RBM33 or empty vector (EV) control. For each sample, enrichment was first normalized to input and then normalized to *GAPDH* in FH-RBM33 pull-downs. (*F*) Pearson correlations between the indicated transcript features and RNA localization in RBM33-deficient cells. (SE) Splicing efficiency. (*G*,*H*) Linear regression analysis showing the correlation between GC content and transcript localization (*G*) or expression (*H*) in *RBM33* KO cells relative to WT cells. (*I*) UCSC genome browser tracks showing CpG islands and GC content of nuclear-enriched transcripts in *RBM33* KO cells. The plot at the *left* is a zoomed-in region of *B* showing the most nuclear-enriched transcripts. Data are represented as mean ± SD with individual data points shown. For qRT-PCR experiments, *n* = 3 biological replicates, and *P*-values were calculated by two-way ANOVA (*D*) or Student's *t*-test (*E*). (**) *P* ≤ 0.01, (****) *P* ≤ 0.0001.

To identify potential RBM33 substrates, we performed whole-transcriptome sequencing (RNA-seq) on nuclear and cytoplasmic RNA from *RBM33* wild-type and knockout cells (Fig. 5A). This revealed a large set of transcripts that exhibited nuclear enrichment in RBM33-deficient cells, including 327 RNAs that showed a greater than twofold increase in their nuclear–cytoplasmic ratios (Fig. 5B; Supplemental Table S3). Notably, *NORAD* was among the most nuclear-enriched transcripts in this data set (rank 40 out of 10,111 transcripts detected). Examination of transcript biotypes revealed that the vast majority of transcripts that exhibited RBM33-dependent export were protein-coding, with modest enrichment of a few noncoding RNA classes (Fig. 5C). Gene ontology analysis demonstrated that transcripts encoding transmembrane proteins, particularly membrane transporters, were enriched among RNAs that rely on RBM33 for nuclear export (Supplemental Fig. S8A). Nuclear–cytoplasmic fractionation followed by qRT-PCR validated the nuclear enrichment of several transcripts in RBM33-depleted cells (Fig. 5D). Furthermore, UV-RIP experiments demonstrated that RBM33 bound directly to *STMN3* and *MAFG*, transcripts whose export was highly dependent on RBM33 (Fig. 5E). These data document the existence of a cohort of transcripts whose nuclear export requires RBM33.

To identify the features that specify RBM33-dependent nuclear export, linear regression analysis was used to identify transcript characteristics that correlated with nuclear enrichment in *RBM33* knockout cells. Since *NORAD* is unspliced, we initially expected that dependency on RBM33 for nuclear export would correlate with exon number or splicing efficiency. Nevertheless, various characteristics related to splicing, including exon number, exon length, exon density, or splicing efficiency, exhibited only weak, albeit statistically significant, correlations with RBM33 dependency (Fig. 5F). Moreover, as a class, spliced transcripts were more dependent on RBM33 for nuclear export compared with intronless RNAs (Supplemental Fig. S8B). In addition, transcripts that depend on RBM33 for nuclear export did not exhibit altered splicing efficiency in *RBM33* knockout cells (Supplemental Fig. S8C), indicating that nuclear trapping was not a secondary consequence of impaired splicing.

In contrast to splicing-related parameters, GC content correlated strongly with nuclear retention in RBM33-deficient cells (Fig. 5F,G). Furthermore, consistent with our finding that nuclear trapping of *NORAD* in cells depleted of RBM33 leads to *NORAD* degradation by the nuclear exosome, we observed that GC content also correlated with reduced transcript abundance in *RBM33* knockout cells (Fig. 5H). In accordance with these results, we observed high GC content throughout the gene bodies of *STMN3*, *MAFG*, and *CBX6*, transcripts whose export is highly dependent on RBM33 (Fig. 5I). Interestingly, although *NORAD* is not a GC-rich transcript overall, a segment with high GC content is present at its 5′ end, suggesting that local GC-rich regions may be sufficient to specify RBM33-dependent export. Indeed, sequences from this region of *NORAD* were previously shown to be sufficient to confer NXF1-dependent export to an unspliced reporter transcript (Zuckerman et al. 2020).

### RBM33 binds to GC-rich elements in nuclear export substrates

To identify direct substrates of RBM33 throughout the transcriptome, as well as to determine the sequence characteristics of RBM33 binding sites, we performed eCLIP with FH-RBM33 (Van Nostrand et al. 2016). Conditions were established for cross-linking of FH-RBM33 to RNA in both HCT116 and HEK293T cells (Supplemental Fig. S9A), and eCLIP experiments were performed in both cell lines.

**Figure 6.**
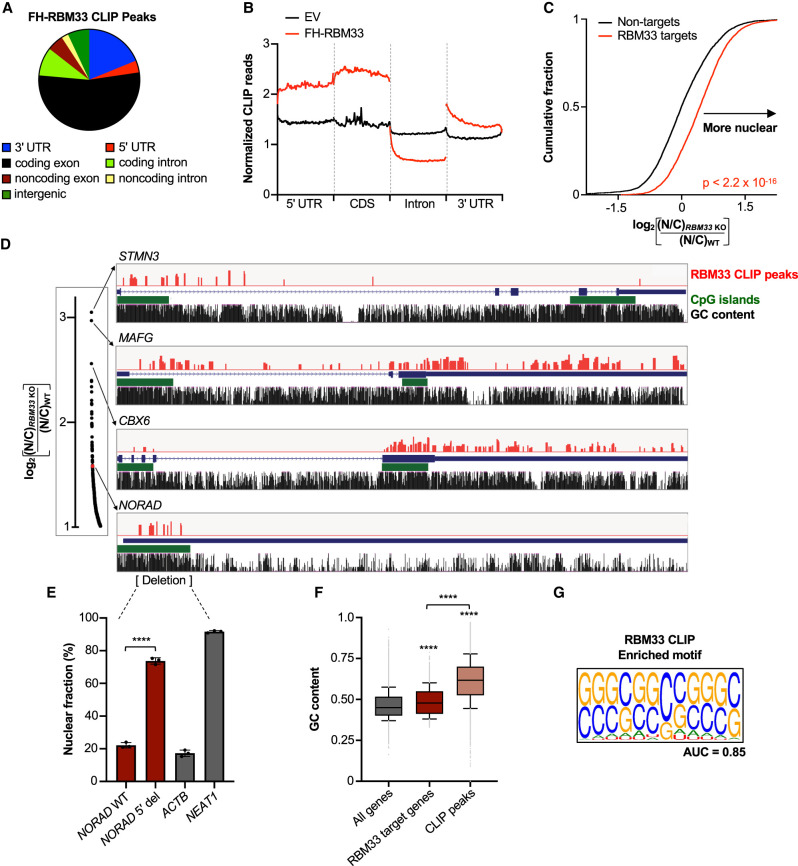
RBM33 binds directly to GC-rich elements in its nuclear export substrates. (*A*) Distribution of RBM33 CLIP peaks. (*B*) Metagene plot showing normalized RBM33 CLIP reads relative to input along the scaled gene body of RBM33 targets in HCT116 cells. (*C*) Cumulative distribution function (CDF) plot comparing the nuclear enrichment of RBM33 CLIP targets with nontargets in *RBM33* KO versus WT HCT116 cells. *P*-value was calculated by two-sample Kolmogorov–Smirnov test. (*D*) The positions of RBM33 CLIP peaks (red) in selected transcripts are shown alongside UCSC genome browser tracks depicting CpG islands and GC content. Only peaks detected in both HCT116 and HEK293T cells are shown. The plot at the *left* is a zoomed-in region of Figure 5B showing the most nuclear-enriched transcripts in *RBM33* KO cells. (*E*) qRT-PCR analysis of nuclear localization of WT *NORAD* or *NORAD* with a 5′ deletion that removes the RBM33 binding sites. Data are represented as mean ± SD with individual data points shown. (****) *P* ≤ 0.0001, calculated by Student's *t*-test (*n* = 3 biological replicates). (*F*) Box plots of GC content. Whiskers represent the 10–90 percentile range, boxes represent the 25–75 percentile range, and outliers are shown as gray dots. (****) *P* ≤ 0.0001, calculated by Wilcoxon rank sum test. (*G*) The consensus motif identified by GraphProt in RBM33 CLIP peaks. AUC (area under curve) is a metric of prediction performance (range 0–1).

RBM33 binding was primarily detected within exons of protein-coding and noncoding RNAs (inclusive of 5′ and 3′ untranslated regions [UTRs]) (Fig. 6A; Supplemental Table S4). Among protein-coding transcripts, RBM33 binding was most enriched within coding sequences, followed by 5′ and 3′ UTRs (Fig. 6B; Supplemental Fig. S9B). Notably, RBM33 binding was depleted from introns, suggesting a preference for binding fully processed, export-competent transcripts, in line with its function as a nuclear export factor. Importantly, RBM33 CLIP targets exhibited preferential nuclear enrichment in RBM33-deficient cells, further establishing that RBM33 promotes the nuclear export of transcripts to which it binds (Fig. 6C). Accordingly, we detected RBM33 CLIP peaks in all examined RBM33-dependent export substrates, including *STMN3*, *MAFG*, *CBX6*, and *NORAD* (Fig. 6D).

Notably, the RBM33 binding sites in *NORAD* were clustered at its GC-rich 5′ end, in the vicinity of sequences that are sufficient to promote NXF1-dependent export of unspliced reporter transcripts (Zuckerman et al. 2020). To test whether these binding sites are required for *NORAD* nuclear export, CRISPR/Cas9 genome editing with paired sgRNAs was used to create a cell line with heterozygous deletion of this RBM33 binding region, enabling examination of wild-type and mutant *NORAD* transcripts in the same cells. Whereas full-length *NORAD* was efficiently trafficked to the cytoplasm, deletion of the RBM33 binding site resulted in nuclear trapping to an extent that closely mirrored the effect of depleting RBM33 (Fig. 6E; Supplemental Fig. S9C). Thus, a GC-rich RBM33 binding site at the 5′ end of *NORAD* is required for efficient nuclear export of this lncRNA.

We next examined the sequence characteristics of RBM33 binding sites throughout the transcriptome. In keeping with our finding that GC content correlates with dependency on RBM33 for nuclear export (Fig. 5F,G), we observed that RBM33-bound transcripts were enriched in GC content, with an even greater enrichment of GC percentage in RBM33 CLIP peaks (Fig. 6F). Moreover, unbiased motif discovery identified a GC-rich sequence as the most enriched motif in RBM33 CLIP peaks (Fig. 6G). Supporting the functional importance of RBM33 binding, RBM33 CLIP peaks displayed increased conservation compared with exons, introns, or gene bodies (Supplemental Fig. S9D). In sum, these data demonstrate that RBM33 recognizes GC-rich elements in its target transcripts and promotes cytoplasmic trafficking by recruiting components of the TREX–NXF1 nuclear export machinery.

## Discussion

Cells use multiple independent features in transcribed RNAs to select those destined for nuclear export. For example, the presence of a 5′ cap, completion of splicing, and association of 3′ end processing factors are among the most well-characterized signals for nuclear export (Katahira 2012; Heath et al. 2016). These features strongly promote recruitment of components of the canonical TREX–NXF1/NXT1 nuclear export pathway, which efficiently transports substrates to the cytoplasm. Previous work has also revealed that GC-rich sequences, particularly at the transcript 5′ end, provide a strong, splicing-independent signal for nuclear export that is dependent on NXF1 (Palazzo et al. 2007; Cenik et al. 2011; Palazzo and Akef 2012; Mordstein et al. 2020; Zuckerman et al. 2020). However, how GC-rich elements selectively engage components of the TREX complex and/or the NXF1/NXT1 heterodimer to promote cytoplasmic trafficking has remained unknown.

In this study, we set out to identify factors that promote the nuclear export of the lncRNA *NORAD*, a 5-kb transcript that is efficiently transported to the cytoplasm despite lacking introns (Lee et al. 2016; Tichon et al. 2016; Elguindy et al. 2019; Kopp et al. 2019). Previous work demonstrated that *NORAD* is exported in an NXF1-dependent manner and showed that GC-rich sequences derived from the *NORAD* 5′ end are sufficient to promote export of an intronless reporter transcript (Zuckerman et al. 2020), but how *NORAD* is recognized by the nuclear export machinery was enigmatic. To investigate this question, we devised a genome-wide screening strategy in which the endogenous *NORAD* transcript was tagged with an IRES-GFP cassette that could only be translated after successful transport to the cytoplasm. Infection of these cells with a genome-wide lentiviral CRISPR library followed by the identification of sgRNAs that were enriched in cells with diminished fluorescence enabled the discovery that RBM33, a poorly characterized RNA binding protein, is essential for *NORAD* nuclear export.

Our subsequent studies of RBM33 demonstrated that this RNA binding protein directs the nuclear export of hundreds of transcripts in addition to *NORAD*. Although we initially expected that the export pathway used by *NORAD* might be specialized for intronless transcripts, we instead found that the vast majority of RBM33 substrates are spliced and are unified by the presence of high GC content. Accordingly, transcriptome-wide binding studies revealed that RBM33 directly recognizes GC-rich elements in its target transcripts. This binding preference likely explains why RBM33 was not detected in previous studies of the *NORAD* interactome, which relied on long-wavelength UV cross-linking of associated proteins to modified uridines (Munschauer et al. 2018). Moreover, more widely used short-wavelength UV cross-linking methods are believed to inefficiently detect interactions of proteins with GC nucleotides (Sugimoto et al. 2012), which may explain the paucity of existing data on the transcriptome-wide binding profile of RBM33. In the future, it will be interesting to investigate whether, in addition to binding to GC-rich sequences, RBM33 has a preference for specific structural features, such as G quadruplexes.

**Figure 7.**
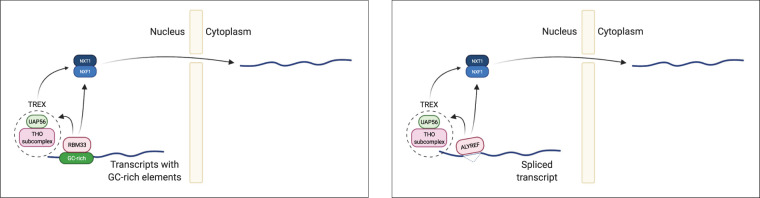
Proposed model for RBM33-dependent nuclear export. (*Left*) RBM33 directs nuclear export by directly binding to GC-rich elements in its target transcripts and recruiting the TREX complex (via UAP56) and the NXF1 nuclear export receptor (along with its obligate partner NXT1). (*Right*) This mechanism parallels the activity of ALYREF, which associates with spliced transcripts and also recruits TREX components and NXF1.

Beyond binding directly to target transcripts, RBM33 interacts with the TREX component UAP56 and export receptor NXF1, thereby promoting the assembly of export-competent ribonucleoproteins (RNPs) (Fig. 7). Interestingly, the canonical RNA export factor ALYREF, which plays a central role in the splicing-dependent export pathway and also interacts with UAP56 and NXF1 (Golovanov et al. 2006), is not required for *NORAD* export. Furthermore, we observed that efficiently spliced transcripts were actually exported more efficiently in the absence of RBM33 (as indicated by a negative correlation of splicing efficiency with nuclear retention in RBM33-deficient cells) (Fig. 5F). These observations suggest that RBM33-dependent export and canonical splicing-dependent export may compete for access to components of the TREX–NXF1/NXT1 pathway. Identification of the domains of RBM33 and UAP56/NXF1 that interact with one another may clarify the relationship between RBM33-dependent and splicing-dependent export, as well as the mechanism by which export of *NORAD* bypasses a requirement for ALYREF.

The discovery of RBM33-mediated export provides a mechanistic basis for the long-standing observation that GC-rich elements, especially when located at the 5′ end of a transcript, enhance cytoplasmic RNA localization and increase gene expression. For example, the nucleotide sequences encoding signal sequences (signal sequence coding regions [SSCRs]), which direct proteins to the endoplasmic reticulum for membrane insertion or secretion, are GC-rich elements that promote nuclear export (Palazzo et al. 2007; Cenik et al. 2011). These sequences resemble RBM33 binding sites and, indeed, transcripts that encode membrane proteins were enriched among those that exhibited impaired nuclear export in RBM33-deficient cells. More generally, it is well established that increasing GC content promotes protein production from various classes of transcripts, including transgenes and viral mRNAs (Kotsopoulou et al. 2000; Kudla et al. 2006; Bauer et al. 2010). Accordingly, recoding transgenes with increased GC content is a frequently used approach to optimize mammalian transgenes for improved expression (Fath et al. 2011). Although it is expected that codon optimization will result in multiple, independent positive effects on gene expression, including enhanced mRNA stability and translation (Hanson and Coller 2018), the GC content of recoded unspliced GFP transgenes was recently shown to strongly correlate with the efficiency of nuclear export (Mordstein et al. 2020). We anticipate that further study of RBM33-mediated export may allow the design of discrete, modular GC-rich nuclear export elements—perhaps consisting of concatemers of RBM33-binding motifs—that may further improve transgene optimization strategies.

RBM33 homologs are present in many vertebrates, while *NORAD* conservation is limited to mammals. Therefore, we speculate that *NORAD* evolved a GC-rich element at its 5′ end, distinct from its PUMILIO binding sequences, to access an existing RBM33-dependent pathway to enable efficient cytoplasmic localization. Unlike *NORAD*, however, most RBM33 substrates are multiexon transcripts. Notably, the feature with the second highest correlation with RBM33-dependent export was low splicing efficiency, although the correlation was much weaker than that associated with GC content. It is therefore likely that many inefficiently spliced RBM33 substrates use both splicing-dependent and RBM33-dependent pathways to achieve adequate cytoplasmic localization. This possibility emphasizes the need for further investigation of the interplay between splicing-dependent and RBM33-dependent export. For example, regulated RBM33 binding could provide a mechanism to selectively enhance the export of poorly spliced or unprocessed RNAs under selected conditions.

Finally, we note that the screening strategy used here to dissect the mechanism of *NORAD* export can easily be adapted to interrogate the export pathways used by other cytoplasmic coding and noncoding RNAs. It is likely that more uncharacterized nuclear export factors with select clientele remain to be identified. Fluorescent reporter-based genetic screens provide a powerful approach to identify these factors and further illuminate our understanding of this fundamental aspect of gene expression.

## Materials and methods

### Generation of *NORAD-IRES-GFP* reporter cell lines

The site chosen for the insertion of the IRES-GFP cassette into *NORAD* can be targeted by two distinct sgRNAs that recognize opposite strands. Oligonucleotides encoding each of these sgRNAs (Supplemental Table S5) were cloned into pX330 (Addgene 42230). HCT116 cells were seeded at 6 × 10^5^ cells per well of a six-well plate and reverse-transfected with either 1.3 µg of donor plasmid alone or 1.3 µg of donor plasmid and 330 ng of pX330. Medium was changed 24 h later. Ten days after transfection, GFP was detectable only in donor/pX330 cotransfected cells. These GFP-positive cells were collected by sorting, and single-cell clones were generated by limiting dilution. Clones were then genotyped by PCR to confirm the insertion of the IRES-GFP cassette at the *NORAD* locus. Two independent clones, each generated with a different guide and exhibiting homozygous insertion of the IRES-GFP cassette, were chosen for screening.

### Genome-wide CRISPR–Cas9 screening

CRISPR–Cas9-mediated loss-of-function screening was performed as described previously (Zhu et al. 2020). Additional details are in the Supplemental Material.

### Subcellular fractionation

Fractionation was performed as described previously (Lee et al. 2016). Additional details are in the Supplemental Material.

### RNA fluorescent in situ hybridization (RNA FISH)

*NORAD* RNA FISH was performed as described previously (Mito et al. 2016; Elguindy et al. 2019). Additional details are in the Supplemental Material.

### Cloning and expression of FH-RBM33, FH-hnRNPC, and FH-PUM2

Sequences of all primers used for cloning are in Supplemental Table S5. FLAG-HA-tagged ORFs were cloned into pLJM1 (Addgene 91980) using HiFi cloning. Additional details are in the Supplemental Material.

### UV cross-linking and RNA immunoprecipitation (UV-RIP)

HCT116 or HEK293T cells (2 × 10^7^) were transfected with 40 µg of pLJM1 empty vector (EV) or pLJM1 FH-RBM33 plasmid using Fugene HD (Promega). Forty-eight hours after transfection, cells were UV cross-linked at 254 nm (400 mJ/cm^2^). Cells were then scraped in PBS, pelleted, snap-frozen in liquid nitrogen, and stored at −80°C until needed. RIP was performed as described (Elguindy et al. 2019). Additional details are in the Supplemental Material.

### FLAG immunoprecipitation and mass spectrometry

HCT116 cells (6 × 10^7^) stably expressing pLJM1 empty (EV) or FH-RBM33 growing in 15-cm plates were scraped in ice-cold PBS, pelleted, snap-frozen in liquid nitrogen, and stored at −80°C. At the time of immunoprecipitation, cells were lysed in 1 mL of lysis buffer (50 mM Tris-HCl at pH 7.5, 150 mM NaCl, 1% NP-40, 0.1% sodium deoxycholate, 2× protease inhibitor tablet [Roche]) for 30 min on ice and cleared by centrifugation at 14,000*g* for 10 min at 4°C. For each pull-down, 5 µg of FLAG M2 antibody (Sigma) was bound to 50 µL of washed Dynabeads Protein G (Invitrogen). Lysates were then added to antibody-coupled beads along with 20 µg/mL RNase A. Reactions were incubated on a rotating platform for 2 h at 4°C, and then beads were washed five times with 1 mL of wash buffer (50 mM Tris-HCl at pH 7.5, 150 mM NaCl, 0.05% NP-40, 2× protease inhibitor tablet [Roche]). Immunoprecipitated proteins were eluted with 40 µL of elution buffer (36 µL of lysis buffer supplemented with 4 µL of 3× FLAG peptide at 10 mg/mL [Sigma]) with shaking at 1000 RPM for 30 min at room temperature. Each IP was performed in triplicate. Mass spectrometry analysis of eluted proteins was performed at the University of Texas Southwestern Proteomics Core using reverse-phase LC-MS/MS and an Orbitrap Fusion Lumos mass spectrometer. Raw MS data files were analyzed using Proteome Discoverer v2.4 SP1 (Thermo), with peptide identification performed using Sequest HT searching against the human protein database from UniProt.

### RNA fractionation sequencing

WT, *RBM33* KO-1, or *RBM33* KO-2 HCT116 cells (4 × 10^5^) were seeded in six-well plates. For each replicate, 48 h later, one well was subjected to total RNA extraction and one well was subjected to nuclear–cytoplasmic fractionation. Whole-transcriptome RNA-seq libraries from 1 µg of DNase I-treated RNA from each sample were prepared with the TruSeq stranded total RNA LT sample preparation kit (Illumina) and sequenced on a NextSeq 500 (Illumina) with 75-bp single-end reads. Reads were mapped to the human reference genome (GRCh38) using STAR v2.7.1a (Dobin et al. 2013). Annotation of transcripts was based on GENCODE v30 (Frankish et al. 2019). The number of reads for each transcript was calculated using featureCounts (Liao et al. 2014). Fragments per kilobase of transcript per million mapped reads (FPKM) were calculated to represent the normalized read counts for each gene using an in-house script. Only transcripts with a FPKM >1 in total RNA, nuclear RNA, and cytoplasmic RNA were included in the analysis. For each gene *g*, the relative RNA localization was calculated as

$$L_{g}=\log_{2}\frac{FPKM\;KO_{nuc}}{FPKM\;KO_{cyto}}-\log_{2}\frac{FPKM\;WT_{nuc}}{FPKM\;WT_{cyto}}.$$

The relative expression for each gene was calculated as

$$E_{g}=\log_{2}\frac{FPKM\;KO_{total}}{FPKM\;WT_{total}}.$$

FPKM was averaged across three wild-type replicates and four *RBM33* KO replicates (two from each KO clone). The longest isoform was used for each gene. GC content, number of exons, transcript length (exons only), longest exon length, exon density (exonic sequence length/gene length), gene length, and splicing efficiency were calculated from GENCODE v30. Splicing efficiency of an intron was calculated as the ratio of reads overlapping exon–exon junctions over the sum of exon–exon- and exon–intron-spanning reads. Splicing efficiency was only calculated for introns in which all exon–exon and exon–intron junctions were covered by at least 10 junction-spanning reads. Mean splicing efficiency refers to the average splicing efficiency across all introns, whereas maximum or minimum splicing efficiency refers to the splicing efficiency of the most efficiently or least efficiently spliced intron, respectively (Zuckerman et al. 2020). Pearson correlation coefficients between the features of transcripts compared with the fold change of their nuclear to cytoplasmic ratio in *RBM33* KO cells were estimated using R. Gene set enrichment analysis (Subramanian et al. 2005) using a preranked list of transcripts ordered by their nuclear-to-cytoplasmic enrichment in *RBM33* KO cells was used for gene ontology analysis of transcripts that exhibit RBM33-dependent export.

### Enhanced UV cross-linking immunoprecipitation (eCLIP)

Direct RNA targets of RBM33 in HCT116 and HEK293T cells were determined by eCLIP, following a published protocol (Van Nostrand et al. 2016), with library design modified as described previously (Kopp et al. 2019). Additional details are in the Supplemental Material.

Other detailed experimental procedures are in the Supplemental Material.

### Data access

All high-throughput sequencing data generated in this study (CRISPR screening, RNA fractionation sequencing, and eCLIP) have been deposited in GEO (accession no. GEO GSE192378). No custom code was generated in this study.

## Supplementary Material

Supplemental Material
