# Supplementary material for: RBM33 directs the nuclear export of transcripts containing GC-rich elements
Source: Genes Dev. 2022 May 1;36(9-10):550–65. doi: 10.1101/gad.349456.122 (PMC9186391; doi:10.1101/gad.349456.122)
Supplement: Supplemental Material [file supp_36_9-10_550__DC1.html]

RBM33 directs the nuclear export of transcripts containing GC-rich elements — Supplemental Material 

# RBM33 directs the nuclear export of transcripts containing GC-rich elements

## Supplemental Material

- Supplemental\_Table\_S1.xlsx
- Supplemental\_Table\_S2.xlsx
- Supplemental\_Table\_S3.xlsx
- Supplemental\_Table\_S4.xlsx
- Supplemental\_Table\_S5.xlsx
- Supplemental\_Figures.pdf
- Supplemental\_Methods.pdf
