# Supplementary material for: RBM33 directs the nuclear export of transcripts containing GC-rich elements
Source: Genes Dev. 2022 May 1;36(9-10):550–65. doi: 10.1101/gad.349456.122 (PMC9186391; doi:10.1101/gad.349456.122)
Supplement: Supplemental Material [file supp_gad.349456.122_Supplemental_Figures.pdf]

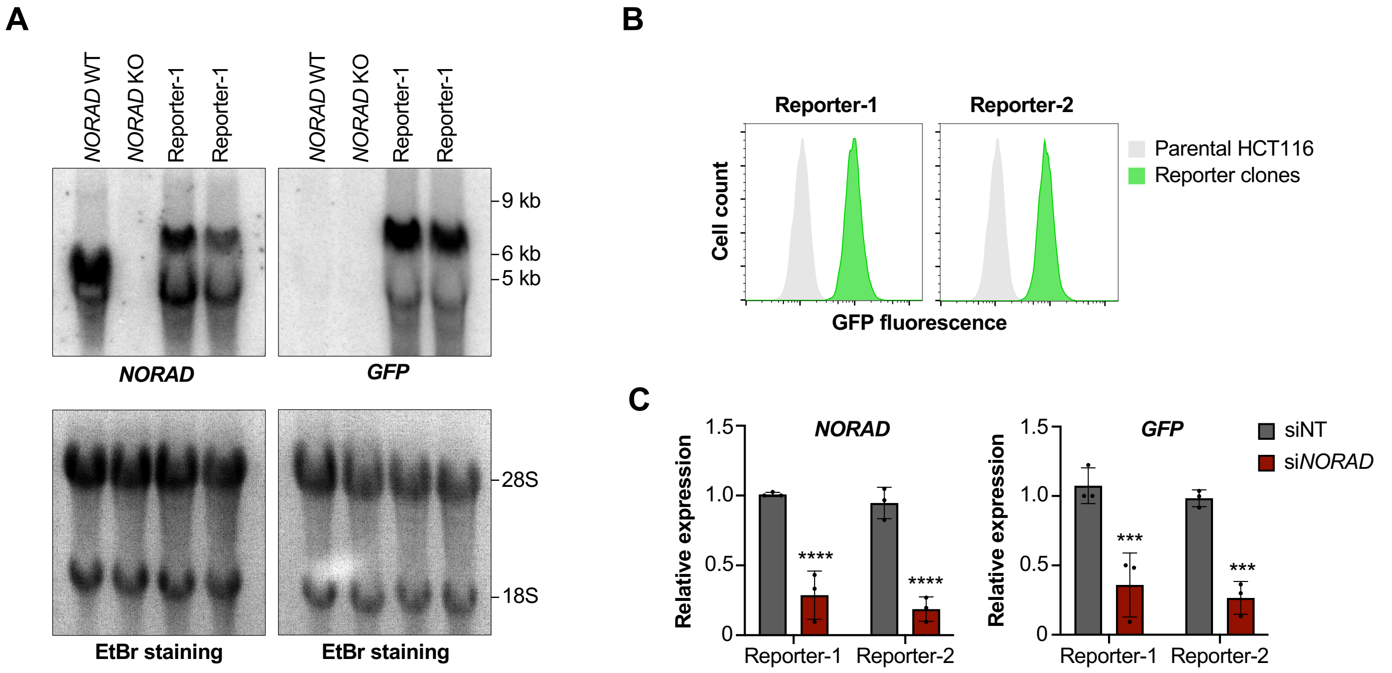

Supplemental Figure S1. Analysis of *NORAD* reporter clones. *(A)* Northern blot analysis of total RNA from the indicated cell lines using probes that detect *NORAD* or *GFP* transcripts. *(B)* Flow cytometry analysis of GFP expression in parental HCT116 cells and *NORAD* reporter clones. *(C)* qRT-PCR analysis of *NORAD* and *GFP* expression relative to 18S rRNA in *NORAD* reporter clones following transfection with non-target siRNA (siNT) or *NORAD*-targeting siRNA (si*NORAD*). Data are represented as mean ± SD with individual data points shown. n=3 biological replicates. ***p≤0.001, ****p≤0.0001; calculated by two-way ANOVA.

A

Ribosome or translation

Gene	Rank	Gene	Rank
PA2G4	2	*UTP3*	25
PCBP2	3	*RIOK1*	27
EIF3B	9	*RPS23*	31
RPS25	11	*DHX33*	32
TRMT112	14	*UTP23*	34
PWP2	15	*RPS17*	35
KRI1	19	*WDR36*	36
IMP3	21	*PTBP1*	37
EMG1	22	*NOB1*	38
UTP14A	24		

Epigenetic modification

Gene	Rank
MORC2	5
FAM208A	8
RTF1	16

Nuclear export

Gene	Rank
NXT1	4
IWS1	7
TPR	18
NUP50	20
THOC6	26
NUP155	33

Cleavage and polyadenylation

Gene	Rank
NUDT21	6
CPSF6	10

Others

Gene	Rank
RBM33	1
ZC3HC1	12
CELF1	13
DDX10	17
SRP14	23
YTHDC1	28
SRSF11	29
NOL7	30

B

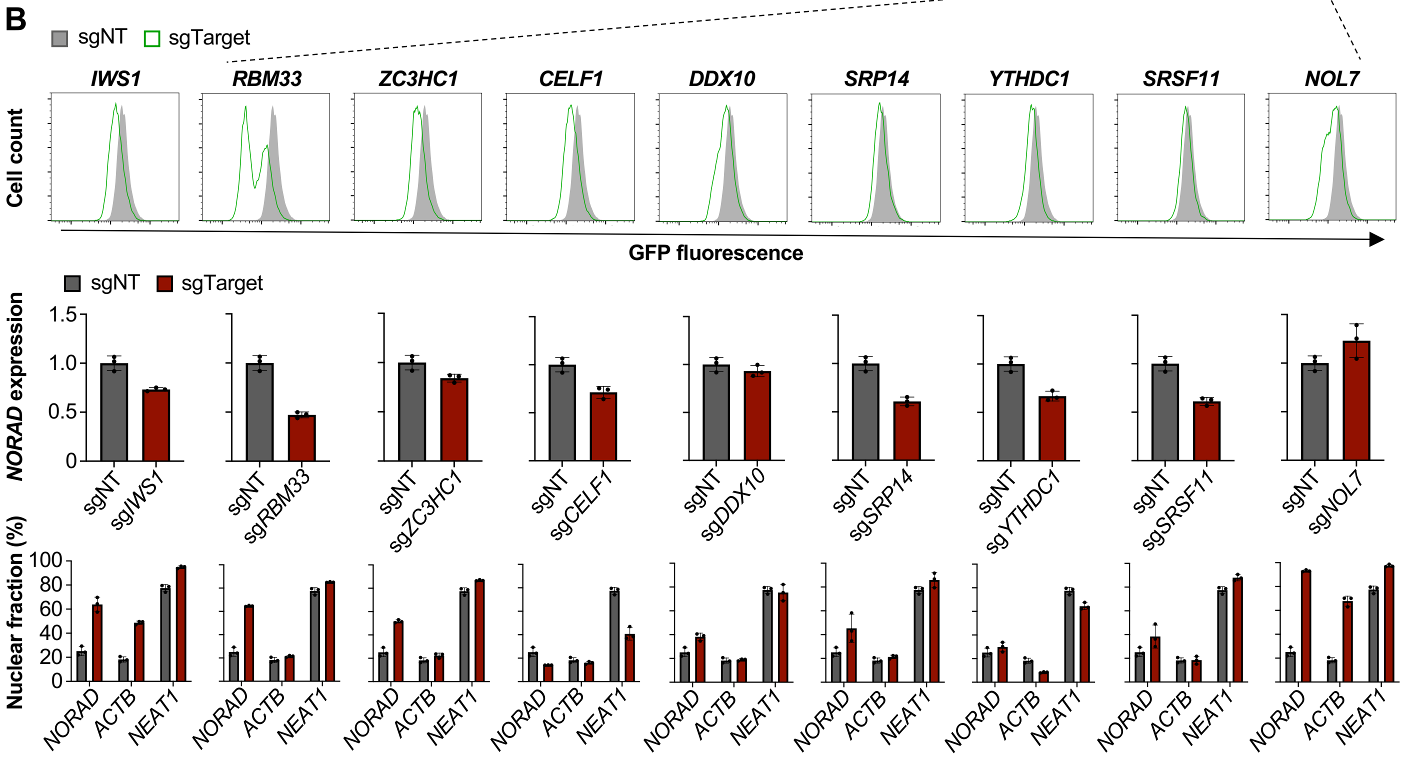

Supplemental Figure S2. Validation of selected hits from the CRISPR-Cas9 screen. *(A)* Functional classification of hits from the CRISPR-Cas9 screen that met the significance threshold of p<0.00001. *(B)* GFP expression (upper row), *NORAD* expression (middle row), and *NORAD*, *ACTB*, and *NEAT1* localization (bottom row) in *NORAD* reporter cells following lentiviral expression of Cas9 and sgRNAs targeting the indicated genes. Mean ± SD from three technical replicates shown.

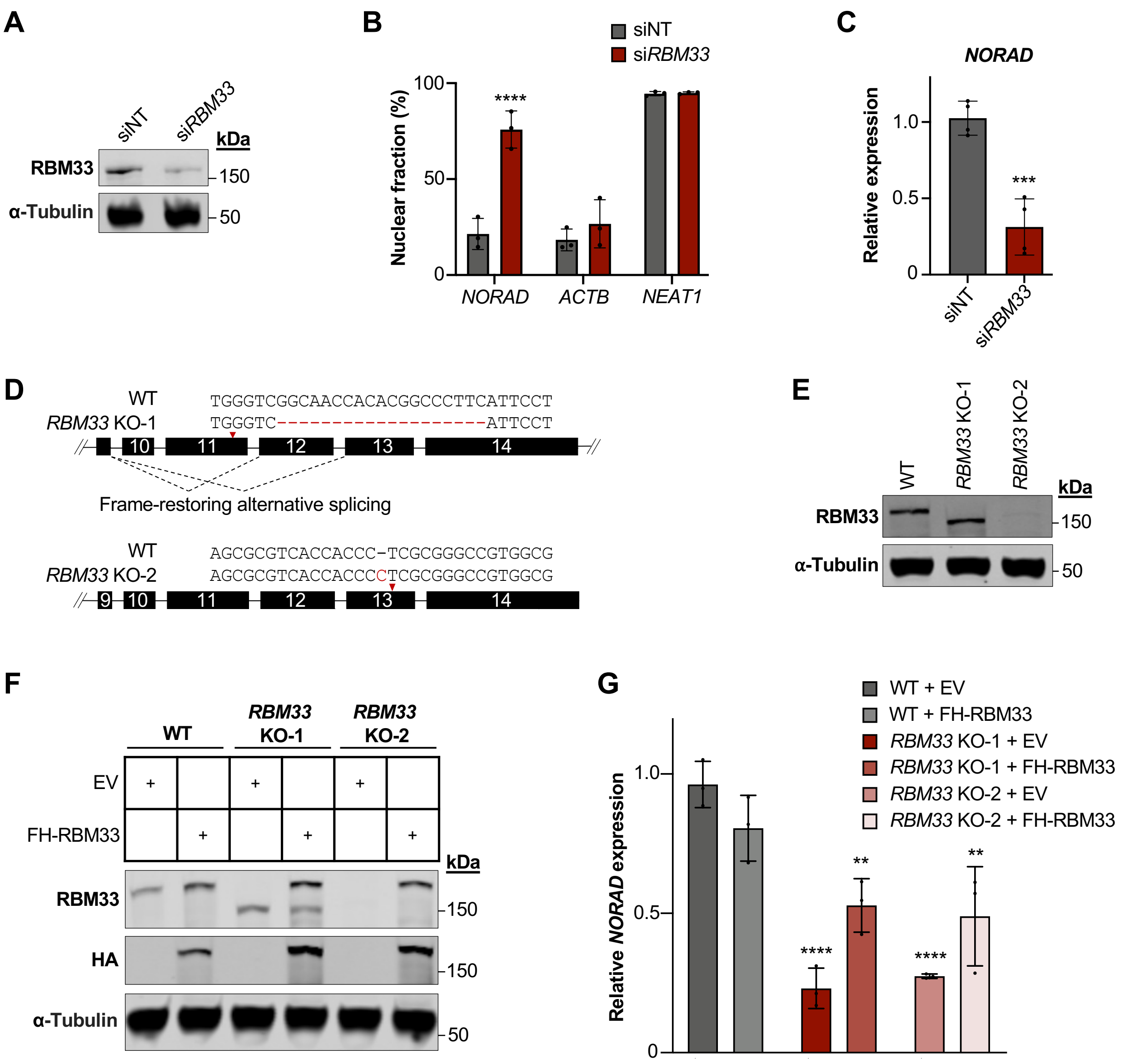

Supplemental Figure S3. Loss of RBM33 results in nuclear enrichment and decreased abundance of *NORAD*. *(A)* Western blot analysis of RBM33 expression in HCT116 cells following treatment with non-target (siNT) or *RBM33*-targeting siRNA Smartpools. α-Tubulin served as loading control. *(B-C)* qRT-PCR analysis of the fraction of *NORAD*, *ACTB*, and *NEAT1* in the nucleus *(B)* or *NORAD* expression relative to 18S rRNA *(C)* following siRNA transfection. *(D)* Sequences of out-of-frame indels (red) generated by CRISPR/Cas9 in two independent *RBM33* KO clones. RT-PCR spanning exons 9-14 demonstrated that *RBM33* KO-1 exhibits frame-restoring alternative splicing of mutant transcripts, as depicted, allowing expression of an internally deleted protein. *(E)* Western blot analysis of RBM33 in parental HCT116 cells and *RBM33* KO clones. *RBM33* KO-1 expresses a shorter isoform of RBM33 due to frame-restoring alternative splicing, as depicted in *(D)*. *(F)* Western blot analysis showing expression of endogenous RBM33 and lentivirally-expressed FH-RBM33. *(G)* qRT-PCR analysis of *NORAD* expression in WT and *RBM33* KO cells following lentiviral expression of FH-RBM33 or EV control. Data are represented as mean ± SD with individual data points shown. For qRT-PCR experiments, n=3 biological replicates and p values were calculated by two-way ANOVA *(B),* student's t-test (*C*), or one-way ANOVA *(G)*. $^{**}p \leq 0.01$, $^{***}p \leq 0.001$, $^{****}p \leq 0.0001$.

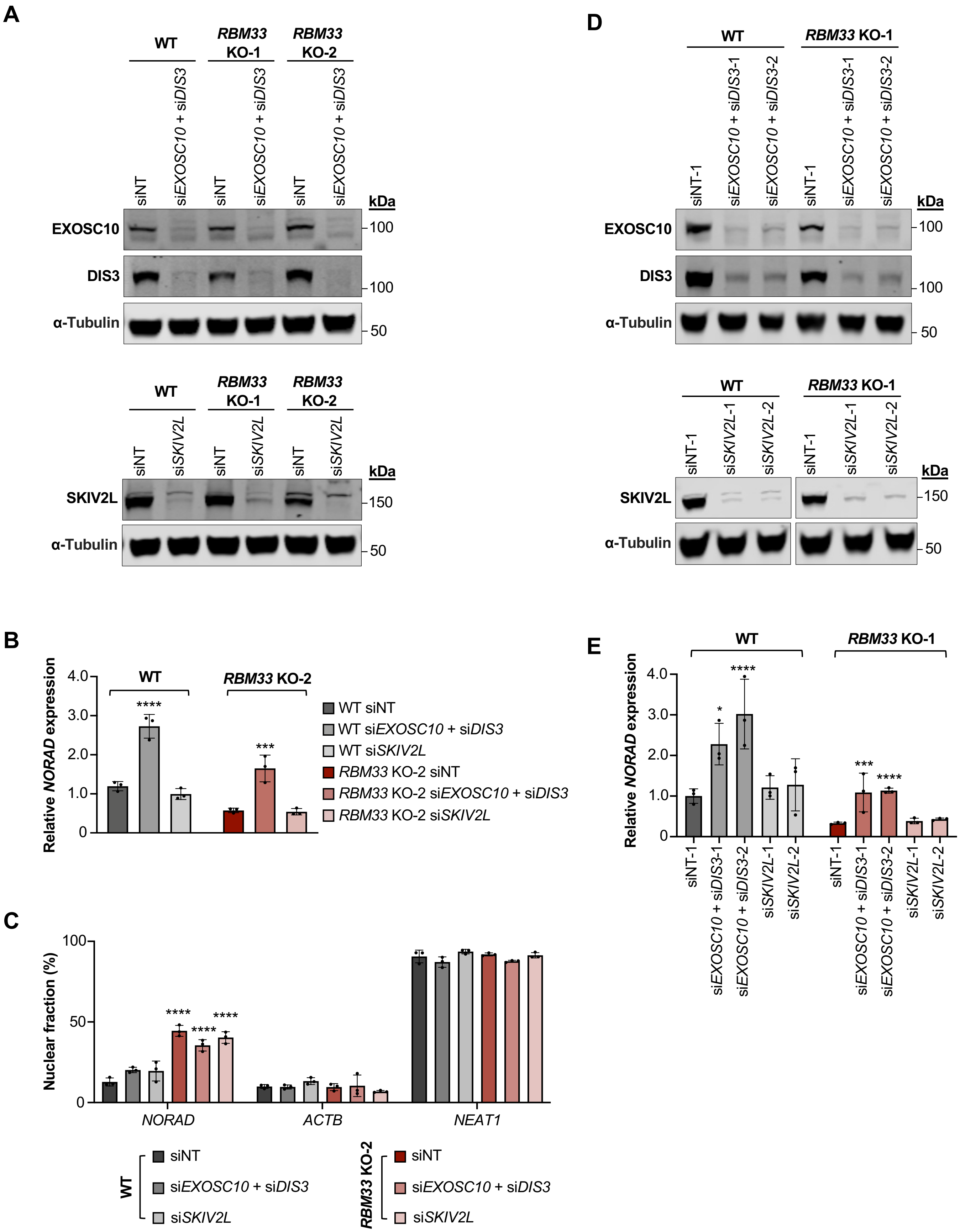
A
WT
RBM33 KO-1
RBM33 KO-2
siNT
siEXOSC10 + siDIS3
kDa
EXOSC10
DIS3
α-Tubulin
100
50
siSKIV2L
SKIV2L
150
B
Relative NORAD expression
WT siNT
WT siEXOSC10 + siDIS3
WT siSKIV2L
RBM33 KO-2 siNT
RBM33 KO-2 siEXOSC10 + siDIS3
RBM33 KO-2 siSKIV2L
C
Nuclear fraction (%)
NORAD
ACTB
NEAT1
D
siNT-1
siEXOSC10 + siDIS3-1
siEXOSC10 + siDIS3-2
siSKIV2L-1
siSKIV2L-2
E

Supplemental Figure S4. Nuclear and cytoplasmic exosome depletion in *RBM33* KO clones. *(A)* Western blot analysis of EXOSC10, DIS3, and SKIV2L expression in WT and *RBM33* KO cells following transfection with siRNA Smartpools. α-Tubulin served as loading control. *(B)* qRT-PCR analysis of *NORAD* expression relative to 18S rRNA in WT and *RBM33* KO-2 cells transfected with the indicated siRNA Smartpools. p values calculated by one-way ANOVA comparing each condition to non-target (NT) siRNA in the same genotype. *(C)* The fraction of *NORAD*, *ACTB*, and *NEAT1* in the nucleus in WT and *RBM33* KO-2 cells transfected with the indicated siRNA Smartpools. p values calculated by two-way ANOVA comparing each condition to siNT in WT cells. *(D)* Western blot analysis of EXOSC10, DIS3, and SKIV2L expression in WT and *RBM33* KO-1 cells following transfection with two independent siRNAs per target. *(E)* qRT-PCR analysis of *NORAD* expression relative to 18S rRNA in WT and *RBM33* KO-1 cells following siRNA transfection. p values calculated by one-way ANOVA comparing each sample to the corresponding siNT control in the same genotype. Data are represented as mean ± SD with individual data points shown. n=3 biological replicates. *$p \leq 0.05$, ***$p \leq 0.001$, ****$p \leq 0.0001$.

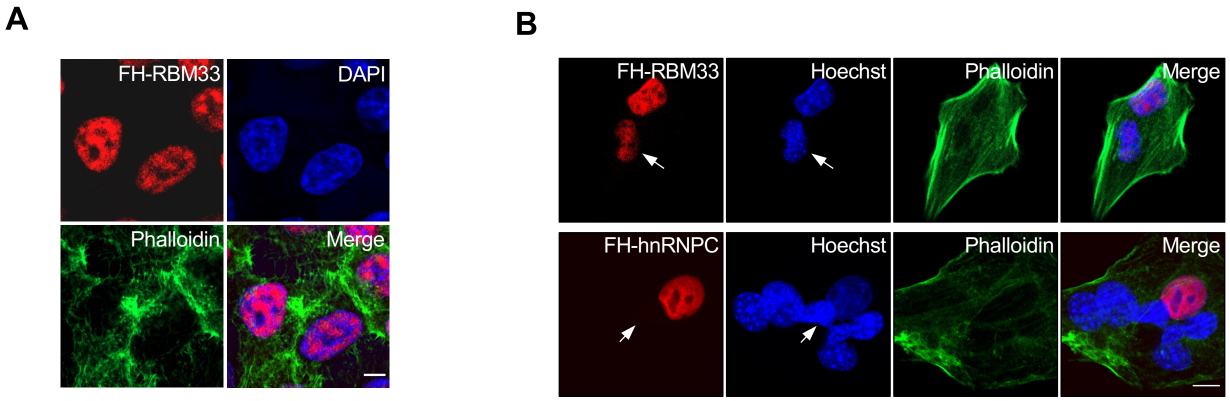

Supplemental Figure S5. Analysis of RBM33 localization and nucleocytoplasmic shuttling. *(A)* Localization of FH-RBM33 in HCT116 cells. Red, FH-RBM33; green, phalloidin; blue, DAPI. Scale bar = 5 μm. *(B)* Heterokaryons were formed from FH-RBM33- or FH-hnRNPC-expressing HCT116 cells and NIH3T3 cells in the presence of cycloheximide to block new protein synthesis, and the localization of FH-RBM33 or FH-hnRNPC was determined by anti-HA immunostaining. FH-hnRNPC served as a negative control in this experiment. Red, anti-HA; green, phalloidin; blue, Hoechst 33258. Mouse nuclei were identified by the distinct Hoechst staining pattern (indicated by white arrows). Scale bar = 10 μm.

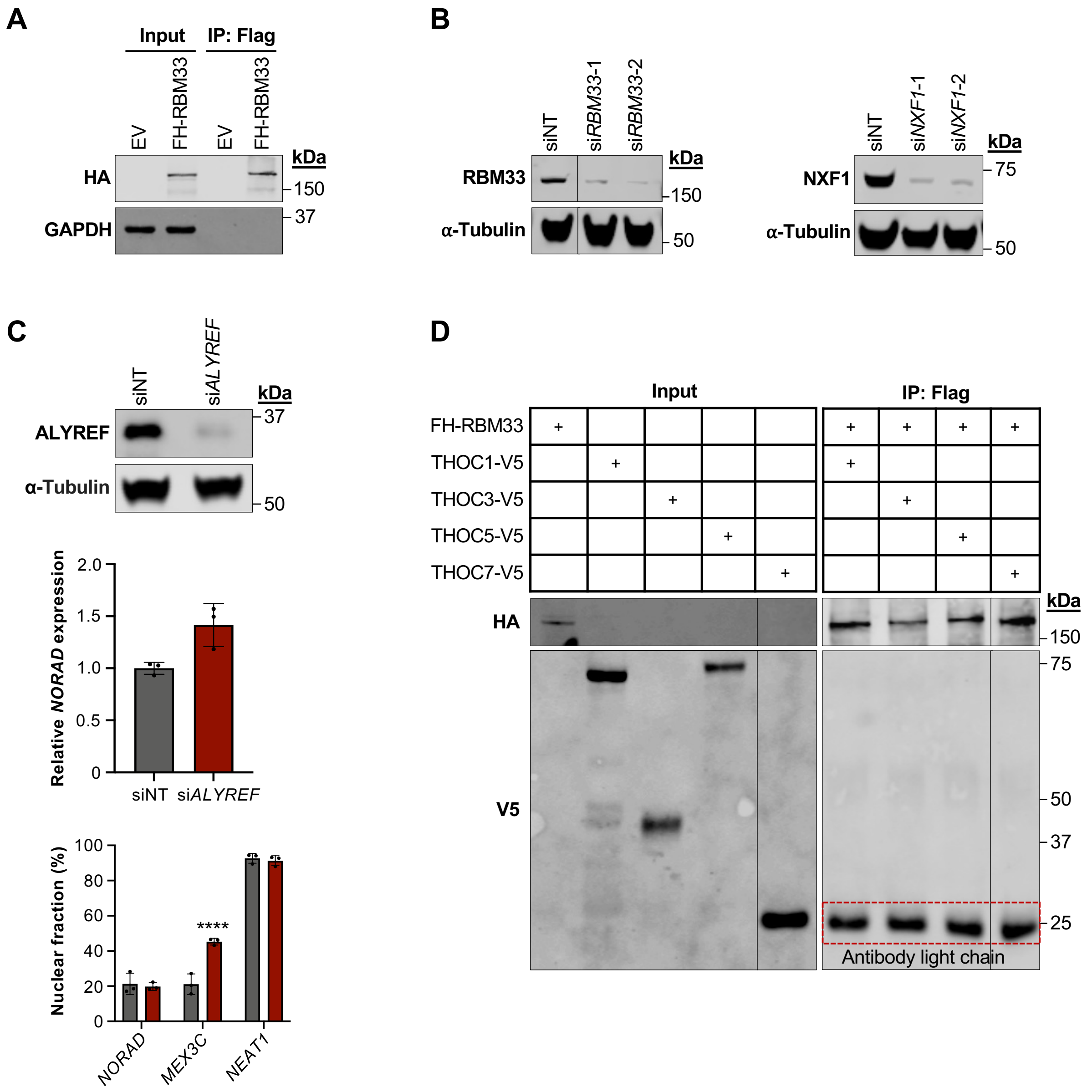

Supplemental Figure S6. Analysis of the role of TREX-NXF1 components in *NORAD* nuclear export. *(A)* Representative western blot of FH-RBM33 in UV-RIP samples used for qRT-PCR analysis in Figure 4B. *(B)* Western blot analysis of RBM33 and NXF1 expression in HCT116 cells following siRNA transfection. α-Tubulin served as loading control. (*C*) Western blot analysis of ALYREF expression (upper), qRT-PCR analysis of *NORAD* expression (middle) and *NORAD*, *MEX3C*, and *NEAT1* localization (lower) in HCT116 cells following transfection with *ALYREF*-targeting siRNA. *MEX3C*, whose export is ALYREF-dependent, served as positive control. p value calculated by two-way ANOVA. Data are represented as mean ± SD with individual data points shown. n=3 biological replicates. ****$p \leq 0.0001$. *(D)* Co-immunoprecipitation of in vitro translated FH-RBM33 and V5-tagged THO subcomplex components detected by western blot. Vertical lines indicate removal of irrelevant lanes.

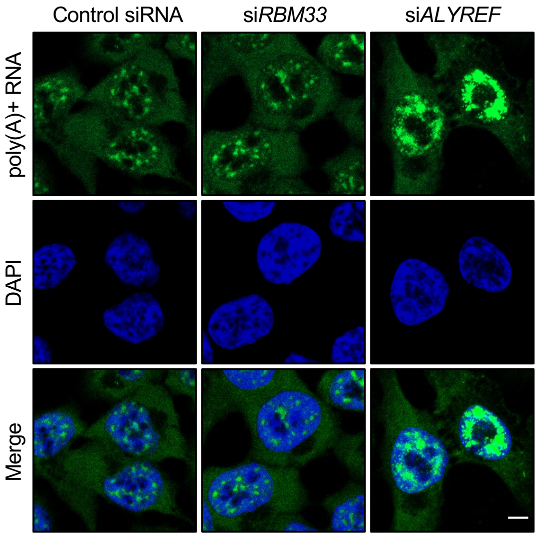

Supplemental Figure S7. RBM33-deficiency does not impair bulk poly(A) RNA export. RNA FISH using a FAM-oligo(dT)50 probe in HCT116 cells transfected with the indicated siRNA Smartpools. Green, poly(A)+ RNA; blue, DAPI. Scale bar = 5 µm.

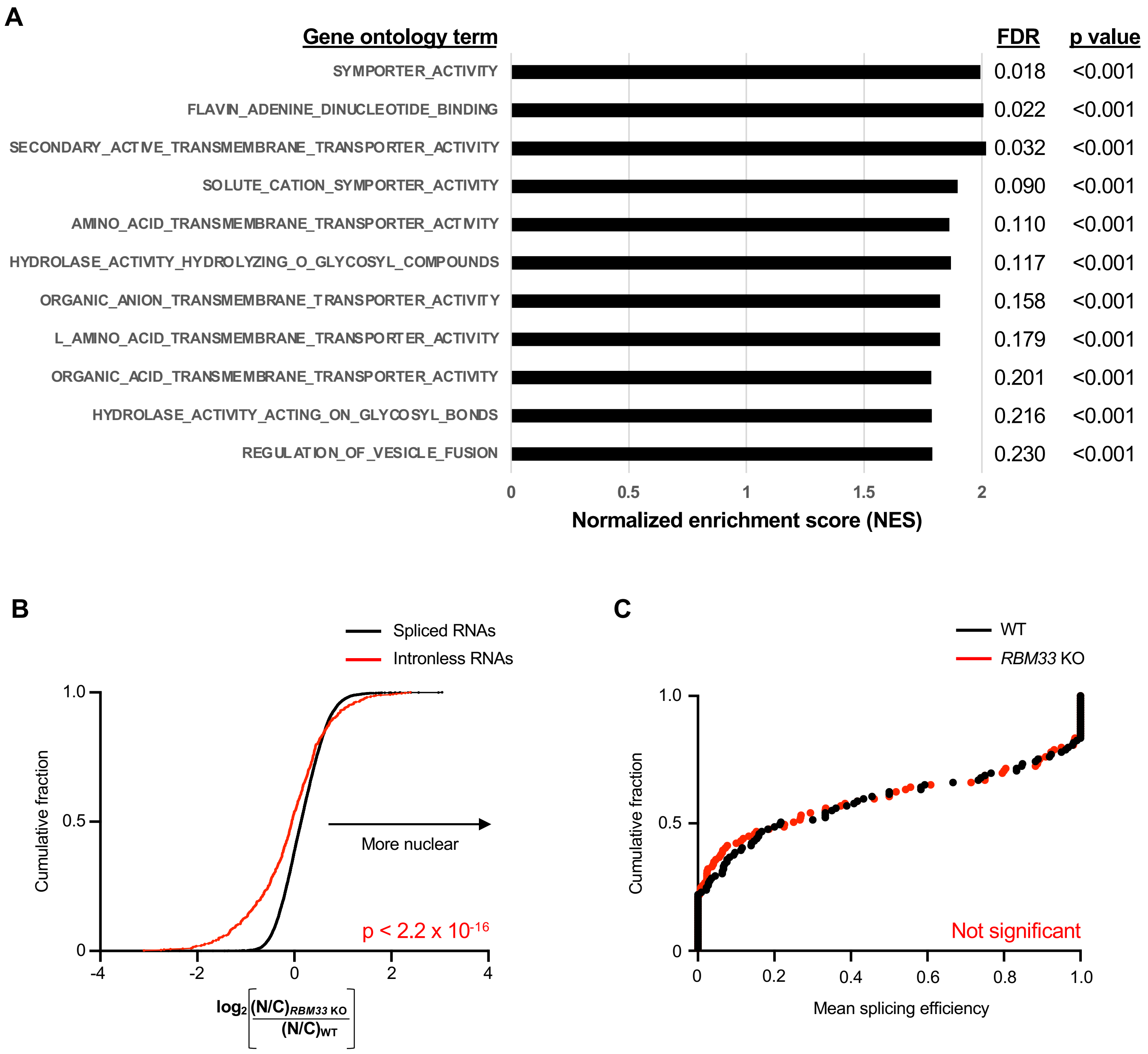

Supplemental Figure S8. Analysis of fractionation-RNA-seq data. *(A)* Gene set enrichment analysis showing nuclear enrichment of transcripts encoding transmembrane transporters in *RBM33* KO cells. Gene ontology gene sets were used for the analysis (C5 collection of the Molecular Signatures Database) and all gene sets showing an FDR<0.25 are shown in the figure. *(B)* Cumulative distribution function (CDF) plot comparing the nuclear enrichment of intronless transcripts to spliced RNAs in *RBM33* KO cells. *(C)* CDF plot comparing the splicing efficiency of transcripts that depend upon RBM33 for nuclear export in *RBM33* WT and KO cells. 109 transcripts that displayed at least two-fold nuclear enrichment in *RBM33* KO cells and exhibited sufficient read coverage to estimate splicing efficiency were used in this analysis. p values for CDF plots calculated by two-sample Kolmogorov-Smirnov test.

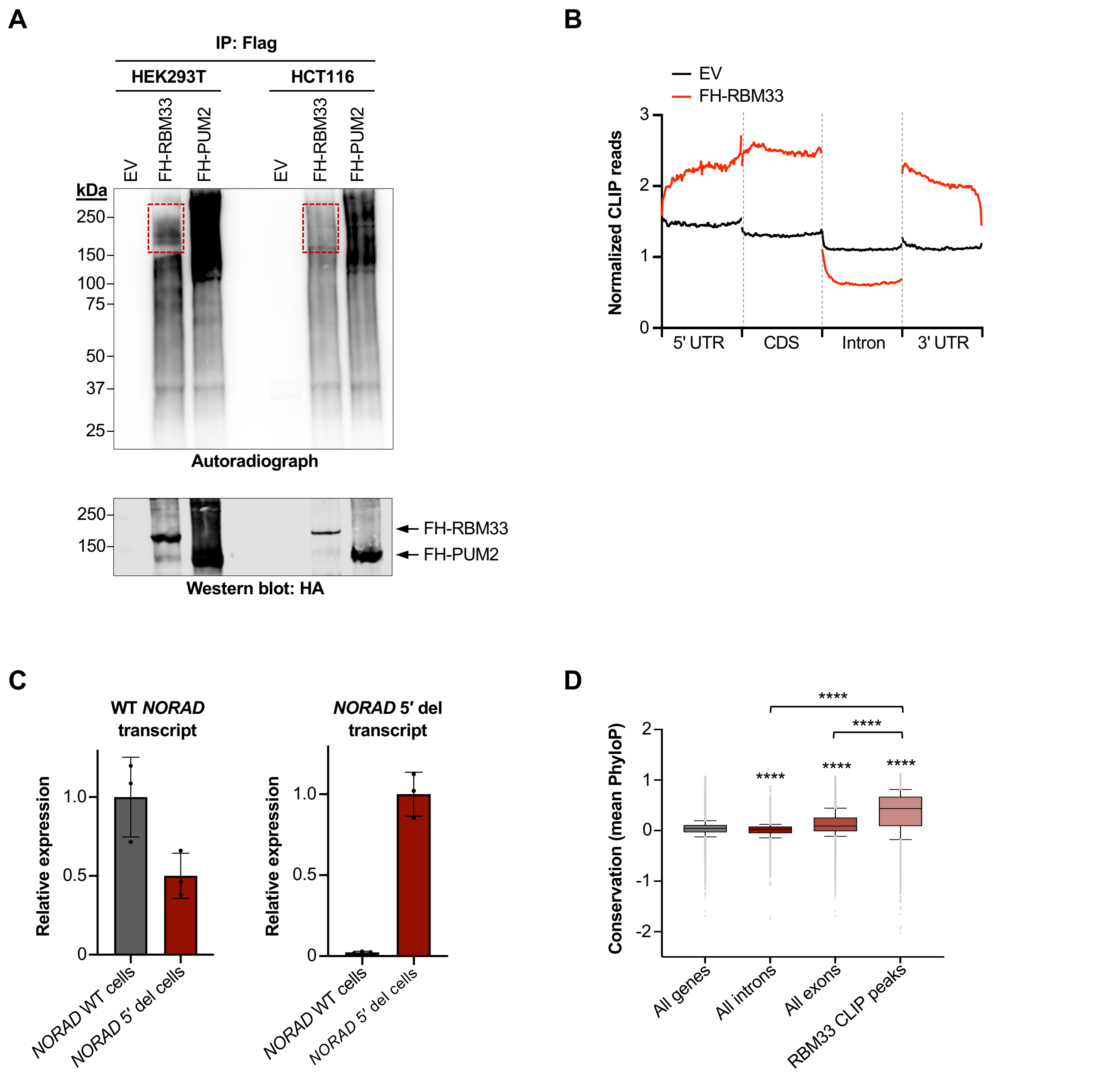

Supplemental Figure S9. RBM33 eCLIP analysis. *(A)* Autoradiograph showing radiolabeled RNAs in complex with FH-RBM33 or FH-PUM2 in HEK293T and HCT116 cells (top). Western blot of FH-RBM33 and FH-PUM2 shows the efficiency of immunoprecipitation in the same samples (bottom). FH-PUM2 served as a positive control in these experiments. Red boxes show regions of nitrocellulose membrane excised for RNA isolation. *(B)* Metagene plot showing normalized RBM33 CLIP reads relative to input along the scaled gene body of RBM33 targets in HEK293T cells. *(C)* qRT-PCR analysis of *NORAD* expression in WT cells or in a cell line harboring a heterozygous deletion of the RBM33 binding region of *NORAD* (5′ del). Primers that specifically detect the WT *NORAD* transcript (left) or the transcript with the 5′ deletion (right) were used. *(D)* Box plots of PhyloP scores. Whiskers represent the 10-90 percentile range with outliers shown as grey dots. ****p≤0.0001; calculated by Wilcoxon rank-sum test.
