# Supplementary material for: RBM33 directs the nuclear export of transcripts containing GC-rich elements
Source: Genes Dev. 2022 May 1;36(9-10):550–65. doi: 10.1101/gad.349456.122 (PMC9186391; doi:10.1101/gad.349456.122)
Supplement: Supplemental Material [file supp_gad.349456.122_Supplemental_Methods.pdf]

SUPPLEMENTAL MATERIALS AND METHODS

Cell culture

Cell lines were obtained from ATCC. HCT116 cells were cultured in McCoy's 5A media (Thermo Fisher Scientific) supplemented with 10% fetal bovine serum (Gibco, Sigma-Aldrich) and 1X Antibiotic-Antimycotic (Gibco). HEK293T cells were grown in Dulbecco's Modified Eagle's Medium (DMEM) (Invitrogen) supplemented with 10% fetal bovine serum (Gibco, Sigma-Aldrich) and 1X Antibiotic-Antimycotic (Gibco). Cell lines were confirmed to be free of mycoplasma contamination.

Construction of *NORAD-IRES-GFP* donor vector

An encephalomyocarditis virus (EMCV) IRES-EGFP cassette was PCR amplified from pCAGIG_V5-PPP6C (Addgene #92013). Left and right *NORAD*-targeting homology arms were PCR amplified from HCT116 genomic DNA. Primers used for all amplifications are provided in Supplemental Table S5. The donor vector backbone was generated from AAVS1 hPGK-PuroR-pA donor (Addgene #22072) by excising PGK-Puro and the *AAVS1* homology arms using SpeI and NotI restriction sites. All four fragments (left and right *NORAD* homology arms, IRES-GFP, and vector backbone) were then assembled in a single NEBuilder HiFi reaction using the manufacturer's protocol and sequence verified.

Genome-wide CRISPR-Cas9 screening

CRISPR-Cas9 mediated loss of function screening was performed as described previously (Zhu et al. 2020). Briefly, the Brunello lentiCRISPR_v2 library (Addgene #73179) was used. The two independent reporter cell lines served as biological replicates. For each replicate, 1.9 x 10^7 cells per dish were seeded into seven 15 cm dishes in media containing 8 µg/mL of polybrene (Millipore) and lentiviral library at a multiplicity of infection of ~0.5. Two days post-transduction, cells were seeded into media containing 1 µg/mL puromycin (Thermo Fisher Scientific). Cells

were selected in puromycin containing media for the next ten days, during which they were passaged every two days. At least 5 x 10^7 cells were seeded at each passage to maintain >500X library coverage. On day 10 in puromycin, the dimmest 0.5% cells were sorted to collect at least 2 x 10^5 cells per replicate, while at least 5 x 10^7 unsorted cells were collected per replicate.

Genomic DNA was isolated from unsorted cells using the MasterPure Complete DNA Purification kit (Lucigen) and from sorted cells by phenol-chloroform extraction as described (Golden et al. 2017). Sequencing libraries were generated using two sequential rounds of PCR using Herculase II Fusion DNA polymerase (Agilent). In the first round of PCR, 6.6 µg of genomic DNA from unsorted cells was used in each of forty 100 µl reactions, and all recovered genomic DNA from sorted cells was split into two 100 µl reactions. Following 18 cycles of PCR1 amplification, 5 µl from the pooled reaction product was used in the second round of PCR (13 – 14 cycles), using primers containing barcodes and Illumina sequencing adaptors (Supplemental Table S5). Amplicons were purified using AMPure XP beads (Beckman Coulter Life Sciences) and sequenced on an Illumina NextSeq500 with 75 bp single-end reads. Approximately 2.4 x 10^7 reads were obtained from each replicate. MAGeCK analysis was used to identify genes targeted by sgRNAs enriched in the sorted versus unsorted populations (Li et al. 2014).

CRISPR-Cas9 mediated gene knockout

sgRNAs targeting genes of interest or control sgRNAs (Supplemental Table S5) were cloned into lentiCRISPR_v2 (Addgene #52961) as previously described (Sanjana et al. 2014). For initial validation of hits in knockout pools (Supplemental Fig. S2B), *NORAD-IRES-GFP* reporter cells transduced with the lentiCRISPR_v2 vectors were selected in media containing 1 µg/ml puromycin for 7 days before sorting to enrich for cells dimmer than the control population for analysis of RNA levels and localization. To generate *RBM33* KO clones, HCT116 cells were transfected with pX458 (Addgene #48138) expressing Cas9, GFP, and sgRNAs targeting

RBM33. 48 hours after transfection, cells were sorted to collect the brightest 10% of cells. Single cell clones were then generated from this pool by limiting dilution and screened for indels at the targeted locus. To identify the alternatively-spliced RBM33 transcripts that contribute to residual expression of a truncated RBM33 protein in *RBM33* KO-1 (Supplemental Fig. S3D,E), RNA was reverse transcribed with SuperScript IV first strand synthesis system (Thermo Fisher Scientific) and cDNA was PCR amplified and sequenced.

siRNA knockdown

siRNA targeting *NORAD* was purchased from Sigma (targeted sequence provided in Supplemental Table S5) and all other siRNAs were purchased from Dharmacon [siNT: D-001810-10-05 (Smartpool); siNT-1: D-001810-01-20; siRBM33: L-028751-02-0005 (Smartpool); siRBM33-1: J-028751-19-0002; siRBM33-2: J-028751-20-0002; siEXOSC10: L-010904-00-0005 (Smartpool); siEXOSC10-1: J-010904-05-005; siEXOSC10-2: J-010904-07-0005; siDIS3: L-015405-01-0005 (Smartpool); siDIS3-1: J-015405-11-0005; siDIS3-2: J-015405-12-0005; siSKIV2L: L-013435-01-0005 (Smartpool); siSKIV2L-1: J-013435-09-0005; siSKIV2L-2: J-013435-10-0005; siALYREF: L-012078-00-0005 (Smartpool); siNXF1-1: J-013680-10-0005; siNXF1-2: J-013680-11-0005]. HCT116 cells were reverse transfected with siRNA using Lipofectamine RNAiMAX (Thermo Fisher Scientific). In the exosome knockdown experiment (Fig. 3; Supplemental Fig. S4), 100 nM total siRNA was used in each condition (100 nM non-targeting siRNA, 100 nM *SKIV2L*-targeting siRNA, or 50 nM each of *EXOSC10* and *DIS3*-targeting siRNA). For all other experiments, siRNA was used at a final concentration of 20 nM. Media was changed 24 hours post-transfection and cells were collected for analysis 72 hours post-transfection, except in case of *NXF1* knockdown where cells were collected 48 hours post-transfection.

Cloning and expression of FH-RBM33, FH-hnRNPC, and FH-PUM2

Sequences of all primers used for cloning are provided in Supplemental Table S5. FLAG-HA-RBM33 (FH-RBM33) was cloned as two separate fragments. The fragment containing the FLAG and HA tag was synthesized by Genewiz and PCR amplified with overhangs for HiFi cloning. The second fragment was PCR amplified from HCT116 cDNA with overhangs for HiFi cloning. These two fragments were then inserted into EcoRI-digested pLJM1 (Addgene #91980) using NEBuilder HiFi assembly according to the manufacturer's protocol. FH-hnRNPC and FH-PUM2 were PCR amplified from the Invitrogen UltimateORF lite library with overhangs for HiFi assembly into EcoRI-digested pLJM1. Stable expression of FH-RBM33 was achieved by transducing WT or *RBM33* KO HCT116 cells with pLJM1-FH-RBM33 lentivirus. Transduced cells were selected in 1 µg/mL puromycin for six days and then switched to media containing 0.25 µg/mL puromycin before analysis. Transient expression of FH-RBM33, FH-hnRNPC, and FH-PUM2 was achieved by transfecting plasmids into recipient cells with Fugene HD (Promega).

RNA extraction and qRT-PCR

Total RNA was extracted from cells using Trizol and the QIAGEN miRNeasy Mini kit with on-column DNase digestion. cDNA was synthesized from 1 µg of total RNA using the Primescript RT Master Mix (Takara) according to the manufacturer's instructions. SYBR Green PCR master Mix (Applied Biosystems) was used for qPCR reactions. RNA expression levels were normalized to *ACTB* or 18S rRNA using standard curves for each gene. Primer sequences used for qRT-PCR are provided in Supplemental Table S5.

Northern blotting

20 µg total RNA was separated on a 0.7% denaturing agarose gel containing formaldehyde and transferred to Hybond N+ membranes. *NORAD* and *GFP* probes were PCR amplified with primers provided in Supplemental Table S5 and radiolabeled using the Random Primed DNA

Labeling Kit (Roche). Hybridization of probes was performed using UltraHyb (ThermoFisher) according to manufacturer's instructions.

Western blotting

Cell lysates were prepared in RIPA buffer (50 mM Tris-HCl pH 8.0, 150 mM NaCl, 1% NP-40, 0.5% sodium deoxycholate, 0.1% sodium dodecyl sulfate) supplemented with 2X Protease inhibitor cocktail (Roche). Proteins were separated on 4-12 % Bis-Tris NuPAGE gels (Thermo Fisher Scientific) and transferred to nitrocellulose membranes. Blocking and antibody incubations were performed in TBST containing 5% non-fat milk. Blots were imaged using a LI-COR Odyssey imager. The following antibodies were used: RBM33 (Bethyl, A303-926A), α-Tubulin (Sigma, T5168), HA (Cell Signaling, 3724S), EXOSC10 (Bethyl, A303-989A-M), DIS3 (Bethyl, A303-764A-M), SKIV2L (Proteintech, 11462-1-AP), NXF1 (Bethyl, A303-913A), UAP56 (Thermo Fisher Scientific, 14798-1-AP), ALYREF (Bethyl, A302-892A), THOC5 (Bethyl, A302-119A), V5 (Invitrogen, R960-25), GAPDH (Cell Signaling, 2118), anti-mouse secondary (Fisher, NC9744100), and anti-rabbit secondary (Fisher, NC9523609).

Subcellular fractionation

Fractionation was performed as described previously (Lee et al. 2016). Briefly, cells were collected by trypsinization and lysed in 175 µL RLN1 buffer [50 mM Tris-HCl pH 8, 140 mM NaCl, 1.5 mM $MgCl_2$, 0.5% NP-40, and 1:1000 SUPERase In RNase inhibitor (Thermo Fisher Scientific)]. Following a 5 minute incubation on ice, the sample was centrifuged at 300 g for 2 minutes. The supernatant, representing the cytoplasmic fraction, was collected and centrifuged at 16000 g for 5 minutes to clear debris. The initial pellet, representing the nuclear fraction, was gently resuspended in 500 µL RLN1 and centrifuged at 1000 g for 5 minutes to wash. The pellet was then resuspended in 175 µL RLN2 buffer [50 mM Tris-HCl pH 8, 500 mM NaCl, 1.5 mM $MgCl_2$, 0.5% NP-40, and 1:1000 SUPERase In RNase inhibitor (Thermo Fisher Scientific)]. RNA

was extracted from each of these fractions by Trizol in combination with the miRNeasy mini kit (Qiagen) with an on-column DNase digestion.

RNA fluorescent in situ hybridization (RNA FISH)

NORAD RNA FISH was performed as described previously (Mito et al. 2016; Elguindy et al. 2019). Briefly, *NORAD* was detected using DIG-labeled anti-sense RNA probes synthesized by in vitro transcription using a DIG-labeling mix (Roche) and purified using Micro Bio-Spin P-30 chromatography columns (Bio-Rad). Primers used for generating the template for in vitro transcription are provided in Supplemental Table S5. 4 x 10^5 cells were seeded on poly-L-lysine coated coverslips in 6-well plates. Two days later, cells were fixed with 4% paraformaldehyde in PBS for 10 minutes at room temperature, washed with PBS, and permeabilized with 0.5% Triton X-100 for 10 minutes at room temperature. Samples were then washed with PBS and incubated with pre-hybridization buffer (50% formamide, 2X SSC, 1X Denhardt's solution, 10 mM EDTA, 0.1 mg/mL yeast tRNA, 0.01% Tween-20) for 1 hour. 10 ng/µL DIG-labelled *NORAD* probe was diluted in hybridization buffer (pre-hybridization buffer containing 5% dextran sulfate), denatured at 75º C for 10 minutes, and used for hybridization at 55º C for 21 hours. After hybridization, samples were washed, treated with RNase A, and blocked for 1 hour at room temperature with blocking reagent (Roche). Primary antibody incubation was carried out in blocking buffer containing 1:100 mouse anti-DIG antibody (Roche) for 1 hour at room temperature. Following TBST washes, secondary antibody incubation was performed in blocking buffer at room temperature for 1 hour with Cy3 labeled goat anti-mouse IgG antibody (EMD Millipore). Nuclei were stained with DAPI (2.5 µg/ml) for 1 minute, washed again with TBST and coverslips were mounted with SlowFade Diamond Antifade Mountant with DAPI (Thermo Fisher Scientific).

For poly(A)+ RNA FISH, 4 x 10^5 HCT116 cells were seeded per well of a 6-well plate on poly-L-Lysine coated coverslips and reverse transfected with 20 nM non-targeting, *RBM33*-targeting, or *ALYREF*-targeting siRNA using lipofectamine RNAiMAX (Thermo Fisher Scientific).

Media was changed 24 hours later. Three days after transfection, cells were fixed with 4% paraformaldehyde for 15 minutes at room temperature, washed with PBS, and permeabilized with 0.5% Triton X-100 for 10 minutes at room temperature. Following a PBS wash, samples were washed with 2X SSC for 10 minutes at room temperature and then incubated in hybridization buffer (1 mg/mL yeast tRNA, 0.005% bovine serum albumin, 10% dextran sulfate, 25% formamide, 2X SSC) for 1 hour at 37º C. 1 ng/uL FAM-oligo(dT)50 probe in hybridization buffer was denatured at 75º C for 5 minutes and added to samples at 37º C for 20 hours. After hybridization, samples were first washed with pre-warmed 4X SSC for 30 minutes at 37º C and then washed with pre-warmed 2X SSC for another 30 minutes. Following a PBS wash, nuclei were stained with DAPI (2.5 µg/ml) for 1 minute, washed again with PBS, and coverslips were mounted with immu-mount (Thermo Fisher Scientific).

Quantification of chromosome segregation defects

Cells were fixed with 4% paraformaldehyde in PBS for 10 minutes at room temperature, washed with PBS, stained with 2.5 µg/ml DAPI for 1 minute, and mounted using SlowFade Diamond Antifade Mountant with DAPI (Thermo Fisher Scientific). Slides were imaged using a Zeiss AxioObserver Z1 microscope. In each sample, 100 anaphase nuclei were assessed for chromosome segregation defects. The experimenter was blinded to the sample identity during quantification.

Tagging of endogenous RBM33 with mNeonGreen

mNeonGreen2 helix 11 (mNG11) was inserted at the N-terminus of RBM33 through homology directed repair (HDR). HDR donor oligonucleotide and sgRNA sequences were designed as described previously (Cho et al. 2022). In brief, HCT116 cells constitutively expressing mNeonGreen2 helices 1-10 (mNG1-10; Addgene #82610) were nucleofected with Alt-R S.p. Cas9 nuclease V3 (IDT), Alt-R CRISPR-Cas9 sgRNA (IDT), Alt-R Cas9 electroporation (IDT)

enhancer, and Alt-R HDR donor oligonucleotide (IDT) using the SE cell line 4D-nucleofector X kit L (Lonza) and a 4D nucleofector device (Lonza) according to the manufacturer's instructions. 24 hours after nucleofection, the media was replaced. Seven days after nucleofection, mNeonGreen positive cells were collected with a FACS Melody cell sorter (BD Biosciences). 72 hours after sorting, cells were seeded on poly-L-lysine coated glass coverslips. 48 hours later, cells were fixed with 4% paraformaldehyde in PBS for 10 minutes. Samples were washed twice with PBS and incubated in blocking buffer (5% FBS and 0.3% Triton X-100 in PBS) for 1 hour at room temperature. Phalloidin staining was performed for 30 minutes at room temperature in blocking buffer. Cells were then washed with PBS and incubated with DAPI (2.5 µg/ml) for 1 minute, washed once with PBS and mounted with immu-mount (Thermo Fisher Scientific).

Immunofluorescence of FH-RBM33

4 x 10^5 HCT116 cells stably expressing FH-RBM33 were seeded on poly-L-lysine coated coverslips in 6-well plates. Two days later, cells were fixed with 4% paraformaldehyde in PBS for 15 minutes at room temperature, washed with PBS, and permeabilized with 0.3% Triton X-100 for 15 minutes at room temperature. Samples were blocked in blocking buffer (5% FBS and 0.3% Triton X-100 in PBS) for 30 minutes at room temperature. Primary antibody incubation was carried out in blocking buffer containing 1:800 rabbit anti-HA antibody (Cell Signaling Technology) overnight at 4º C. Following three PBS washes of 5 minutes each, secondary antibody incubation was performed with 1:500 goat anti-rabbit IgG (Molecular Probes) in blocking buffer at room temperature for 1 hour, followed by three PBS washes. Phalloidin staining was performed for 20 minutes at room temperature in blocking buffer between the second and third PBS wash after secondary antibody incubation. Nuclei were stained with DAPI (2.5 µg/ml) for 1 minute, washed again with PBS, and then coverslips were mounted with immu-mount (Thermo Fisher Scientific).

Heterokaryon assay

1 x 10^6 WT HCT116 cells were seeded in each of two wells of a 6-well plate. 1 µg of FH-RBM33 or FH-hnRNPC was transfected into distinct wells using Fugene HD (Promega). 24 hours later, 2.5 x 10^5 NIH3T3 cells and 2.5 x 10^5 transfected HCT116 cells were mixed and seeded onto poly-L-lysine coated coverslips in 6-well plates. 24 hours later co-cultures of the cells were incubated with 100 ug/ml cycloheximide (Sigma) for 30 minutes. Cells were then fused using prewarmed (37º C) 50% PEG Hybri-Max (Sigma) for 2 min at 37º C. Coverslips were washed with PBS and cultured with fresh media containing 100 µg/ml cycloheximide. 3 hours later, cells were fixed and imaged as described above for anti-HA immunofluorescence, with the modification that Hoechst 33258 (1 µg/ml) was used to stain for and differentiate between human and mouse nuclei in place of DAPI.

Image acquisition and analysis

All RNA FISH and immunofluorescence images were acquired on a Zeiss LSM980 confocal microscope with a 63x oil objective. Endogenously tagged RBM33 was imaged with a 40x water objective. Images were analyzed using Fiji (ImageJ v1.52p). To quantify the localization of *NORAD* FISH signals, the nuclear boundary was determined using DAPI staining, and a mask was generated to segment the nuclei. All non-nuclear signal was regarded as cytoplasmic. The nuclear mask was inverted to obtain the cytoplasmic signal.

Actinomycin D treatment

4 x 10^5 WT, *RBM33* KO-1, and *RBM33* KO-2 HCT116 cells were seeded per well of a 6-well plate. Two days later, media containing Actinomycin D was added at a final concentration of 5 µg/mL and samples were collected 1 hour, 2 hours, or 4 hours after treatment. The samples were subjected to nuclear-cytoplasmic fractionation as described above. Following RNA extraction with the miRNeasy kit (Qiagen), cDNA was generated using SuperScript IV first

strand synthesis system (Thermo Fisher Scientific) with Oligo dT probes. *NORAD* levels relative to *ACTB* abundance in the cytoplasmic fraction was then determined by qRT-PCR at each time point and normalized to the 1 hour time point.

Generation of a *NORAD* allele with deletion of the RBM33 binding site

4 x 10^5 HCT116 cells were seeded in one well of a 6-well plate and reverse transfected using Fugene HD (Promega) with 1 µg each of two pX458 (Addgene #48138) vectors expressing Cas9, GFP, and an sgRNA targeting opposite ends of the RBM33 binding site in *NORAD*. 48 hours after transfection, cells were sorted to collect the brightest 10% of cells. Single cell clones were then generated from this pool by limiting dilution and screened for deletions at the targeted locus by PCR (primers in Supplemental Table S5). The clone analyzed in Fig. 6E has one WT *NORAD* allele and one *NORAD* allele with the RBM33 binding site deletion. The expression and localization of transcripts generated from each of these alleles was uniquely detected using qRT-PCR primers specific for each allele.

Interaction assays with in vitro transcribed/translated proteins

RBM33 or GFP (bait) was PCR amplified with a FLAG-HA tag at the N-terminus. All other proteins (prey) were PCR amplified with a V5 tag at the C-terminus. Proteins were synthesized in a 10 µL reaction using the TnT quick coupled transcription/translation system (Promega) according to the manufacturer's instructions. For each pulldown, 2 µg FLAG M2 antibody (Sigma) was bound to 20 µL washed Dynabeads Protein G (Invitrogen). Antibody-coupled beads were resuspended in 180 µL binding buffer (10 mM HEPES pH 7.5, 3 mM $MgCl_2$, 100 mM KCl, 5 mM EDTA, 5% glycerol, 0.5 % NP-40) and 9 µL each of bait and prey protein was added. Reactions were incubated on a rotating platform for 1 hour at room temperature and beads were washed five times with 400 µL wash buffer (50 mM Tris-HCl pH 7.5, 150 mM NaCl,

0.05% NP-40, 2x protease inhibitor). Proteins were eluted by boiling the beads in 1X LDS sample buffer (NuPAGE) containing 50 mM DTT.

UV crosslinking and RNA immunoprecipitation (UV-RIP)

2 x 10^7 HCT116 or HEK293T cells were seeded per 15 cm dish and reverse transfected with 40 µg pLJM1 empty vector (EV) or pLJM1 FH-RBM33 plasmid using Fugene HD (Promega). Media was changed 24 hours later. 48 hours after transfection, cells were washed with cold PBS and UV crosslinked on ice at 254 nm (400 mJ/cm2) in a Spectrolinker XL-1500 (Spectronics). Cells were then scraped in PBS, pelleted, snap-frozen in liquid nitrogen and stored at -80º C until needed. RIP was performed as described (Elguindy et al. 2019). Briefly, cells were lysed in 1 mL cold iCLIP lysis buffer (50 mM Tris-HCl, 100 mM NaCl, 1% NP-40, 0.1% SDS, 0.5% sodium deoxycholate, 1:200 Protease Inhibitor Cocktail III, RNase inhibitor) for 30 minutes on ice. Lysates were cleared by centrifugation at 14,000 g for 10 minutes at 4º C and the supernatant was added to 3.75 mg pre-washed Dynabeads Protein G (Invitrogen) coupled with 5 µg FLAG M2 antibody (Sigma). After a 2 hour incubation at 4º C, beads were washed three times with 900 µL cold High Salt Wash Buffer (50 mM Tris-HCl, 1 M NaCl, 1 mM EDTA, 1% NP-40, 0.1% SDS, 0.5% sodium deoxycholate) and three times with 500 µL wash buffer (20 mM Tris-HCl, 10 mM $MgCl_2$, 0.2% Tween-20). The beads were then resuspended in 100 µL wash buffer, 70 µL of which was used for RNA extraction and the rest was used for Western blotting.

UV-RIP autoradiography

UV-RIP was performed as described above until the last step where washed beads were resuspended in 100 µL wash buffer. 20 µL of final bead suspension was then used for western blotting to assess immunoprecipitation efficiency. The protein-crosslinked RNA in the remaining 80 µL was radiolabeled with γ-^{32}P-ATP using T4 polynucleotide kinase (NEB) for 15 minutes at 37º C, washed with 500 µL wash buffer, and eluted by boiling beads in 1.5X LDS sample buffer

(NuPAGE). Samples were then loaded on a 1.5 mm 4-12% Bis-Tris NuPAGE gel and transferred to a nitrocellulose membrane. Labeled protein-RNA complexes were detected using a Typhoon Phosphorimager.

Enhanced UV crosslinking immunoprecipitation (eCLIP)

Direct RNA targets of RBM33 in HCT116 and HEK293T cells were determined by eCLIP, following a published protocol (Van Nostrand et al. 2016), with library design modified as described previously (Kopp et al. 2019). Briefly, 2 x 10^7 HCT116 or HEK293T cells were seeded in a 15 cm dish and reverse transfected with 40 µg pLJM1-EV or pLJM1-FH-RBM33 plasmid using Fugene HD (Promega). Media was changed 24 hours later. 48 hours after transfection, UV crosslinking was performed on ice at 254 nm (400 mJ/cm2) in a Spectrolinker XL-1500 (Spectronics). Cells were scraped in PBS, pelleted, snap-frozen in liquid nitrogen and stored at -80º C until needed. Monoclonal FLAG M2 antibody (F1804, Sigma) conjugated to Dynabeads Protein G (Invitrogen) was used to immunoprecipitate FH-RBM33. For each cell line, duplicate size-matched input and IP samples were prepared and sequenced. Adapters were trimmed using Cutadapt v3.1 (Martin 2011), and UMI information for each read was extracted using UMI-tools v1.1.2 (Smith et al. 2017). The pseudogenes of *NORAD* were masked from the reference genome GRCh38, and reads were mapped to the masked genome using STAR v2.7.1a (Dobin et al. 2013). PCR duplicates were marked based on UMI information using UMI-tools and removed. Only uniquely mapped reads were used for the following analyses. Annotation of the transcripts were based on GENCODE v30 (Frankish et al. 2019). Read depth was calculated for each genomic position using Samtools v1.10 (Li et al. 2009), and the normalized reads per million (RPM) was calculated using an in-house script. For different regions (5' UTR, CDS, intron, and 3' UTR), the RPM ratio (IP/input) was aggregated across all RBM33 target genes for EV and FH-RBM33 respectively to generate the metagene analysis.

A CLIP peak was called if the IP/input RPM ratio was greater than two and detected in both HCT116 and HEK293T FH-RBM33 samples but absent in EV samples. All transcripts with a CLIP peak were considered to be RBM33 targets. For the CDF plot depicting the localization of RBM33 targets in *RBM33* KO versus WT (Figure 6C), the non-targets consisted of all transcripts detected in the RNA fractionation-sequencing analysis that were not CLIP targets. GC-content was calculated for each peak with a length of at least 8 nucleotides. The RBM33 binding motif was identified using GraphProt (Maticzka et al. 2014). FASTA files with the RBM33 CLIP peak sequences were used as the training set for bound sequences. An equal number of randomly selected sequences (mapping to gene bodies) of equal length as the RBM33 CLIP peaks were used as the training set for unbound sequences. PhyloP scores based on 20 mammalian species were downloaded from the UCSC genome browser (Pollard et al. 2010). PhyloP score for each peak with a length of at least 8 nucleotides was calculated as the average PhyloP score across all positions of the peak.

SUPPLEMENTAL REFERENCES

Cho NH, Cheveralls KC, Brunner A-D, Kim K, Michaelis AC, Raghavan P, Kobayashi H, Savy L, Li JY, Canaj H, et al. 2022. OpenCell: Endogenous tagging for the cartography of human cellular organization. *Science* **375**: eabi6983.

Dobin A, Davis CA, Schlesinger F, Drenkow J, Zaleski C, Jha S, Batut P, Chaisson M, Gingeras TR. 2013. STAR: ultrafast universal RNA-seq aligner. *Bioinformatics* **29**: 15-21.

Elguindy MM, Kopp F, Goodarzi M, Rehfeld F, Thomas A, Chang T-C, Mendell JT. 2019. PUMILIO, but not RBMX, binding is required for regulation of genomic stability by noncoding RNA *NORAD*. *eLife* **8**: e48625.

Frankish A, Diekhans M, Ferreira AM, Johnson R, Jungreis I, Loveland J, Mudge JM, Sisu C, Wright J, Armstrong J, et al. 2019. GENCODE reference annotation for the human and mouse genomes. *Nucleic Acids Res* **47**: D766-D773.

Golden RJ, Chen B, Li T, Braun J, Manjunath H, Chen X, Wu J, Schmid V, Chang TC, Kopp F, et al. 2017. An Argonaute phosphorylation cycle promotes microRNA-mediated silencing. *Nature* **542**: 197-202.

Kopp F, Elguindy MM, Yalvac ME, Zhang H, Chen B, Gillett FA, Lee S, Sivakumar S, Yu H, Xie Y, et al. 2019. PUMILIO hyperactivity drives premature aging of *Norad*-deficient mice. *eLife* **8**: e42650.

Lee S, Kopp F, Chang TC, Sataluri A, Chen B, Sivakumar S, Yu H, Xie Y, Mendell JT. 2016. Noncoding RNA *NORAD* Regulates Genomic Stability by Sequestering PUMILIO Proteins. *Cell* **164**: 69-80.

Li H, Handsaker B, Wysoker A, Fennell T, Ruan J, Homer N, Marth G, Abecasis G, Durbin R, 1000 Genome Project Data Processing Subgroup. 2009. The Sequence Alignment/Map format and SAMtools. *Bioinformatics* **25**: 2078-2079.

Li W, Xu H, Xiao T, Cong L, Love MI, Zhang F, Irizarry RA, Liu JS, Brown M, Liu XS. 2014. MAGeCK enables robust identification of essential genes from genome-scale CRISPR/Cas9 knockout screens. *Genome Biol* **15**: 554.

Martin M. 2011. Cutadapt removes adapter sequences from high-throughput sequencing reads. *EMBnet.journal* **17**: 10-12.

Maticzka D, Lange SJ, Costa F, Backofen R. 2014. GraphProt: modeling binding preferences of RNA-binding proteins. *Genome Biol* **15**: R17.

Mito M, Kawaguchi T, Hirose T, Nakagawa S. 2016. Simultaneous multicolor detection of RNA and proteins using super-resolution microscopy. *Methods* **98**: 158-165.

Pollard KS, Hubisz MJ, Rosenbloom KR, Siepel A. 2010. Detection of nonneutral substitution rates on mammalian phylogenies. *Genome Res* **20**: 110-121.

Sanjana NE, Shalem O, Zhang F. 2014. Improved vectors and genome-wide libraries for CRISPR screening. *Nat Methods* **11**: 783-784.

Smith T, Heger A, Sudbery I. 2017. UMI-tools: modeling sequencing errors in Unique Molecular Identifiers to improve quantification accuracy. *Genome Res* **27**: 491-499.

Van Nostrand EL, Pratt GA, Shishkin AA, Gelboin-Burkhart C, Fang MY, Sundararaman B, Blue SM, Nguyen TB, Surka C, Elkins K, et al. 2016. Robust transcriptome-wide discovery of RNA-binding protein binding sites with enhanced CLIP (eCLIP). *Nat Methods* **13**: 508-514.

Zhu X, Zhang H, Mendell JT. 2020. Ribosome Recycling by ABCE1 Links Lysosomal Function and Iron Homeostasis to 3' UTR-Directed Regulation and Nonsense-Mediated Decay. *Cell Rep* **32**: 107895.
